# Protein arginylation targets alpha synuclein, facilitates normal brain health, and prevents neurodegeneration

**DOI:** 10.1038/s41598-017-11713-z

**Published:** 2017-09-12

**Authors:** Junling Wang, Xuemei Han, Nicolae Adrian Leu, Stephanie Sterling, Satoshi Kurosaka, Marie Fina, Virginia M. Lee, Dawei W. Dong, John R. Yates, Anna Kashina

**Affiliations:** 1 University of Pennsylvania, School of Veterinary Medicine, Philadelphia, PA 19104 USA; 2 Institute for Biomedical Informatics, Perelman School of Medicine, University of Pennsylvania, Pennsylvania, USA; 3 The Scripps Research Institute, La Jolla, CA 92037 USA; 4 University of Pennsylvania, Perelman School of Medicine, Philadelphia, PA 19104 USA

## Abstract

Alpha synuclein (α-syn) is a central player in neurodegeneration, but the mechanisms triggering its pathology are not fully understood. Here we found that α-syn is a highly efficient substrate for arginyltransferase ATE1 and is arginylated *in vivo* by a novel mid-chain mechanism that targets the acidic side chains of E46 and E83. Lack of arginylation leads to increased α-syn aggregation and causes the formation of larger pathological aggregates in neurons, accompanied by impairments in its ability to be cleared via normal degradation pathways. In the mouse brain, lack of arginylation leads to an increase in α-syn’s insoluble fraction, accompanied by behavioral changes characteristic for neurodegenerative pathology. Our data show that lack of arginylation in the brain leads to neurodegeneration, and suggests that α-syn arginylation can be a previously unknown factor that facilitates normal α-syn folding and function *in vivo*.

## Introduction

Alpha synuclein (α –syn) is a central player in neurodegeneration. Its abnormal accumulation in the brain underlies the pathology of multiple neurodegenerative disorders, collectively termed synucleinopathies, and constitutes the hallmark feature of Parkinson’s disease (PD) (see, e.g. ref. 1 for a recent review). α-syn is a highly abundant brain protein, normally associated with synaptic transmission. Its pathological accumulation in neurodegeneration involves its misfolding, resulting in abnormal aggregation and eventually in its accumulation as fibrillar aggregates known as Lewy bodies that are commonly seen in the brain of PD patients^2, 3^. The molecular mechanisms that trigger α-syn’s misfolding and lead to the disease pathology are not fully understood.

Protein arginylation is a posttranlsational modification mediated by arginyltransferase ATE1 that transfers Arg from tRNA directly to protein targets^4^. Deletion of Ate1 in mice leads to embryonic lethality and impairments in multiple physiological systems, including cardiovascular development^5^, angiogenesis^6^, muscle contraction^7, 8^, and cell migration^9, 10^. Early studies suggested that ATE1 may play a role in nerve regeneration after injury^11, 12^, may target multiple proteins in the brain^13, 14^, and is able to facilitate removal of pathologically generated protein fragments implicated in neurodegeneration^15^. It has been previously believed that ATE1 targets exclusively the N-terminus of proteolytically generated Asp and Glu, but recent studies showed that the acidic side chains of Asp and Glu can also serve as targets for arginylation in intact proteins^16^. This possibility greatly expands the potential scope of arginylation and its biological effects and has been implicated, e.g., in regulation of skeletal muscle contraction^8^, however the direct biological effects of this side chain modification for a single protein target have never been explored.

Here we found that α-syn constitutes a highly efficient target for mid-chain arginylation and is arginylated *in vivo* on two critical Glu residues, one of them previously implicated in familial cases of neurodegeneration. Lack of arginylation results in intracellular accumulation of α-syn in the cultured cells and in mouse brain, and leads to pronounced symptoms of neurodegeneration. Moreover, arginylation reduces the ability of α-syn seeds added to neuronal culture to form intracellular aggregates. Our study is the first demonstration of physiological regulation of a single protein target by mid-chain arginylation that implicates arginylation as a previously unknown mechanism of maintaining normal brain health and preventing α-syn’s abnormal accumulation in the brain and neurodegeneration.

## Results

### α-syn is an efficient substrate for ATE1 *in vitro*

**Figure 1:**
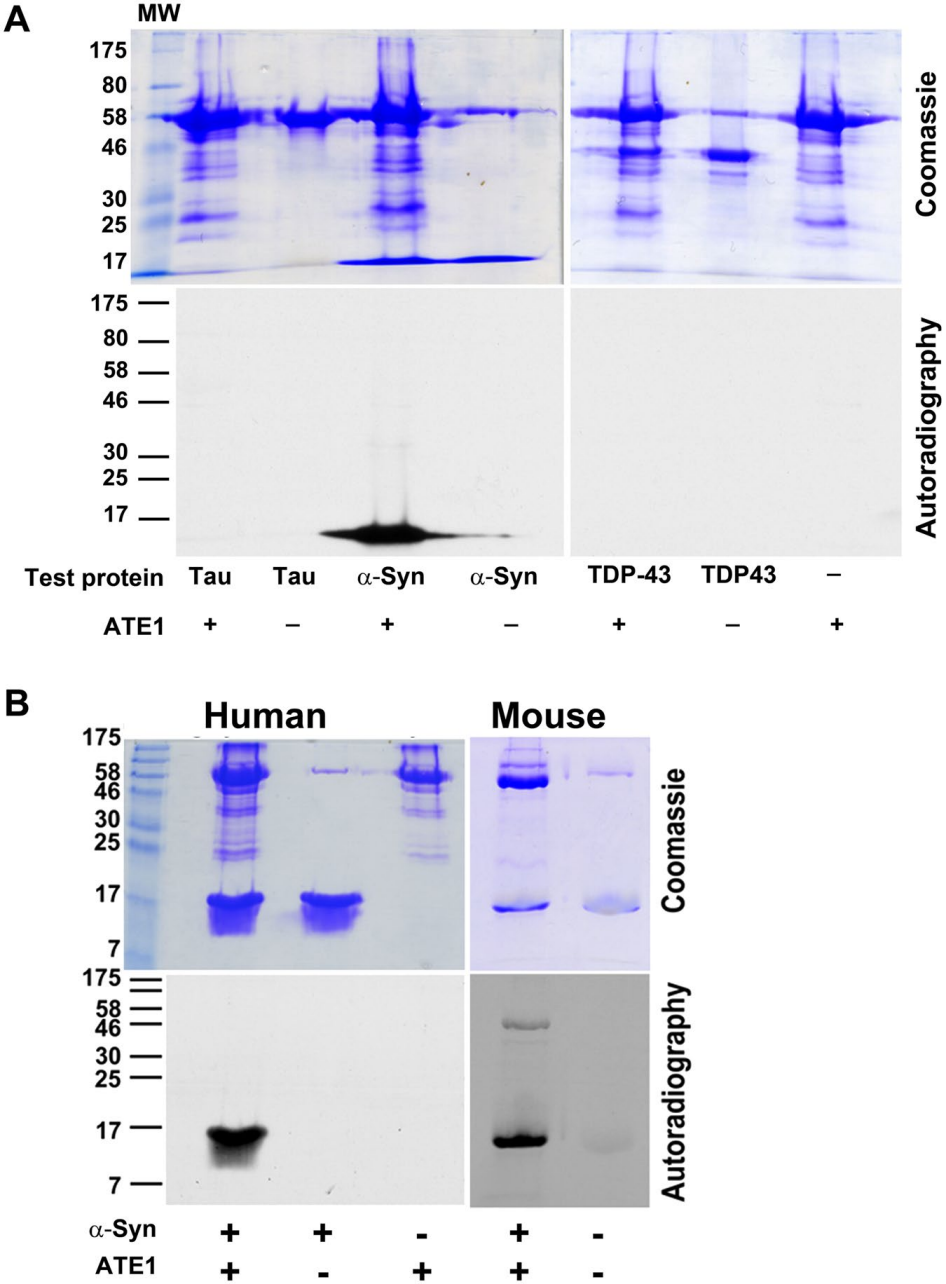
α-syn is arginylated *in vitro*. (**A**) *In vitro* arginylation of purified preparations of human tau, α-syn, and TDP-43 using radioactively labeled [^14^C-Arg] and detected by SDS PAGE (top) and autoradiography (bottom). The lines underneath the lanes list the components added to each reaction. (**B**) *In vitro* arginylation of human and mouse α-syn. Top, Coomassie stained gel. Bottom, autoradiograph.

**Figure 2:**
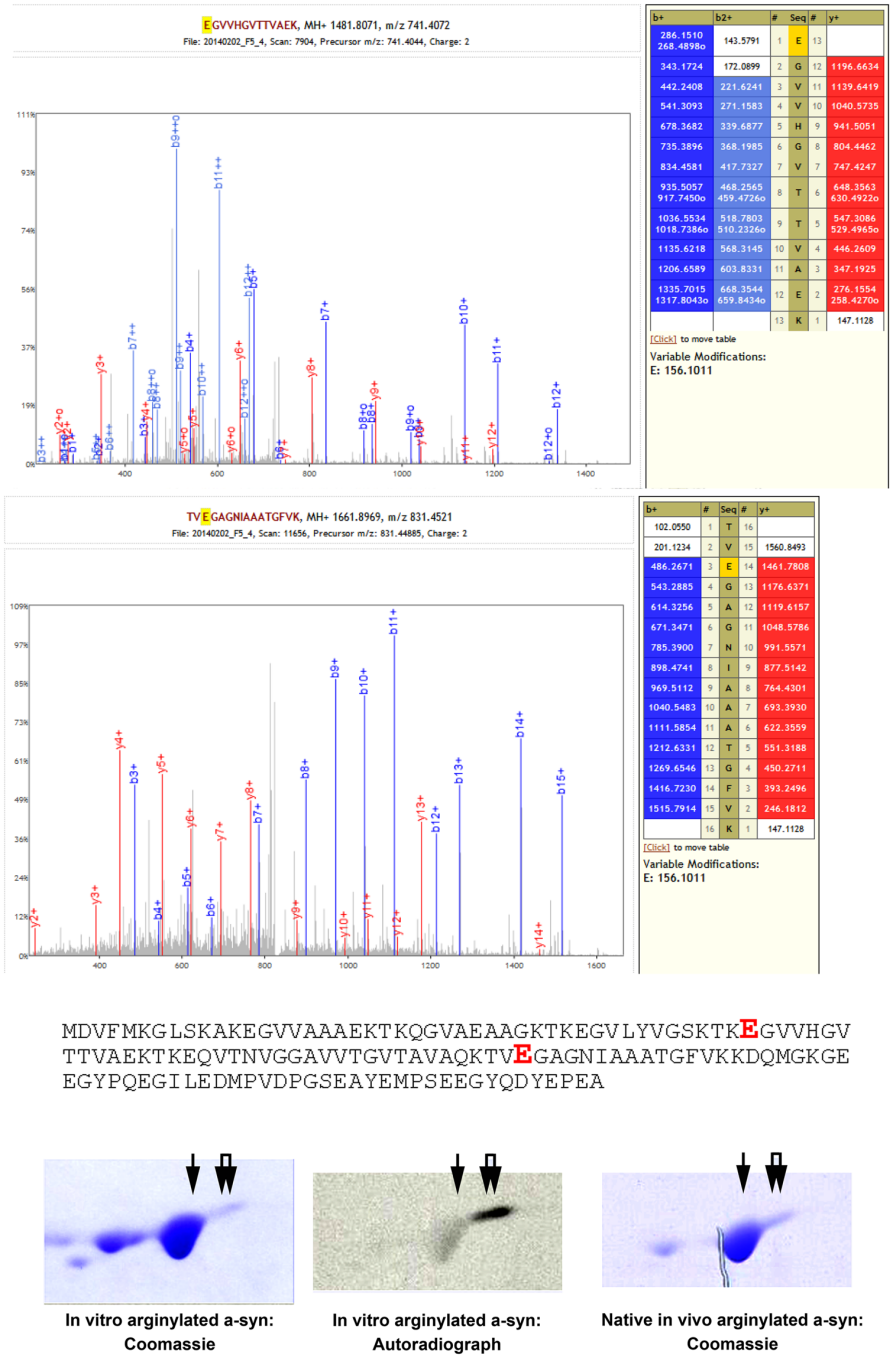
α-syn is arginylated *in vivo*. Top, mass spectra of α-syn peptides arginylated in the mouse brain with the full α-syn sequence underneath. The arginylated residues identified in the native mouse brain are indicated in red enlarged font. Bottom, 2D gel comparison of α-syn preparations arginylated *in vitro* using [^14^C-Arg] (visualized by Coomassie staining, left, and autoradiography, middle) and native preparation of α-syn from the mouse brain (visualized by Coomassie staining, right). Single and double arrows indicate the spots containing arginylated α-syn.

α-syn^17–19^ is an abundant protein in the brain, which can assume different conformations in different physiological context and contains multiple Asp and Glu residues at critically important functional sites, while notably lacking any Arg residues. These features make it a highly interesting potential target for arginylation, since arginylation at Asp and Glu could have dramatic consequences to its function. To test if α-syn can be arginylated, we used our previously developed *in vitro* arginylation assay that utilizes [^14^C-Arg], which can be easily detected by autoradiography^20^. As a control in these assays, we also used two other neurodegeneration-relevant proteins, tau^21, 22^, and TDP43^23–26^. While tau and TDP43 showed no detectable arginylation in these assays, α-syn was very highly arginylated, much more efficiently than any other protein substrate tested to date (Fig. 1A). This high extent of arginylation was not species-specific and was observed using both recombinantly expressed mouse and human α-syn (Fig. 1B), suggesting that ATE1 truly favors α-syn as a substrate. Notably, in these *in vitro* assays arginylation occurred on the full length protein and not proteolytically derived fragments (Figs 1 and 2).

### α-syn is arginylated *in vivo*

To test whether α-syn is arginylated *in vivo* in normal mouse brain, we purified α-syn from the freshly isolated brains of wild type mice and analyzed it by mass spectrometry to detect arginylation using our previously developed method^16, 27, 28^ (see Fig. S1 for the gel of purification fractions). Remarkably, this analysis showed that two internally located E (Glu) residues in α-syn, E46 and E83, were arginylated on their acidic side chains (Fig. 2). Notably, the E46 sites harbors a previously identified mutation E46K implicated in familial PD^29^ and the E83 residue has been previously found to protect α-syn from misfolding^30^. Since the target groups for Arg addition on both sites constitute acidic side chains of the E residues, it appears likely that arginylation happens on intact α-syn *in vivo*, and not on its proteolytic fragments. To confirm this, we compared the 2D electrophoretic mobility of the natively purified α-syn to α-syn arginylated *in vitro* using [^14^C-Arg] (Fig. 2, bottom). The *in vivo* α-syn preparation had similar electrophoretic mobility and spot distribution to this *in vitro* arginylated sample (Fig. 2, bottom). Moreover, in the *in vivo* sample the right-shifted spot corresponding to that with the highest radioactive label *in vitro* (and thus likely to be the pure arginylated species) was even more prominent than *in vitro*, suggesting that the stationary level of this modification in the brain can be reasonably high.

### Lack of arginylation leads to intracellular α-syn accumulation

**Figure 3:**
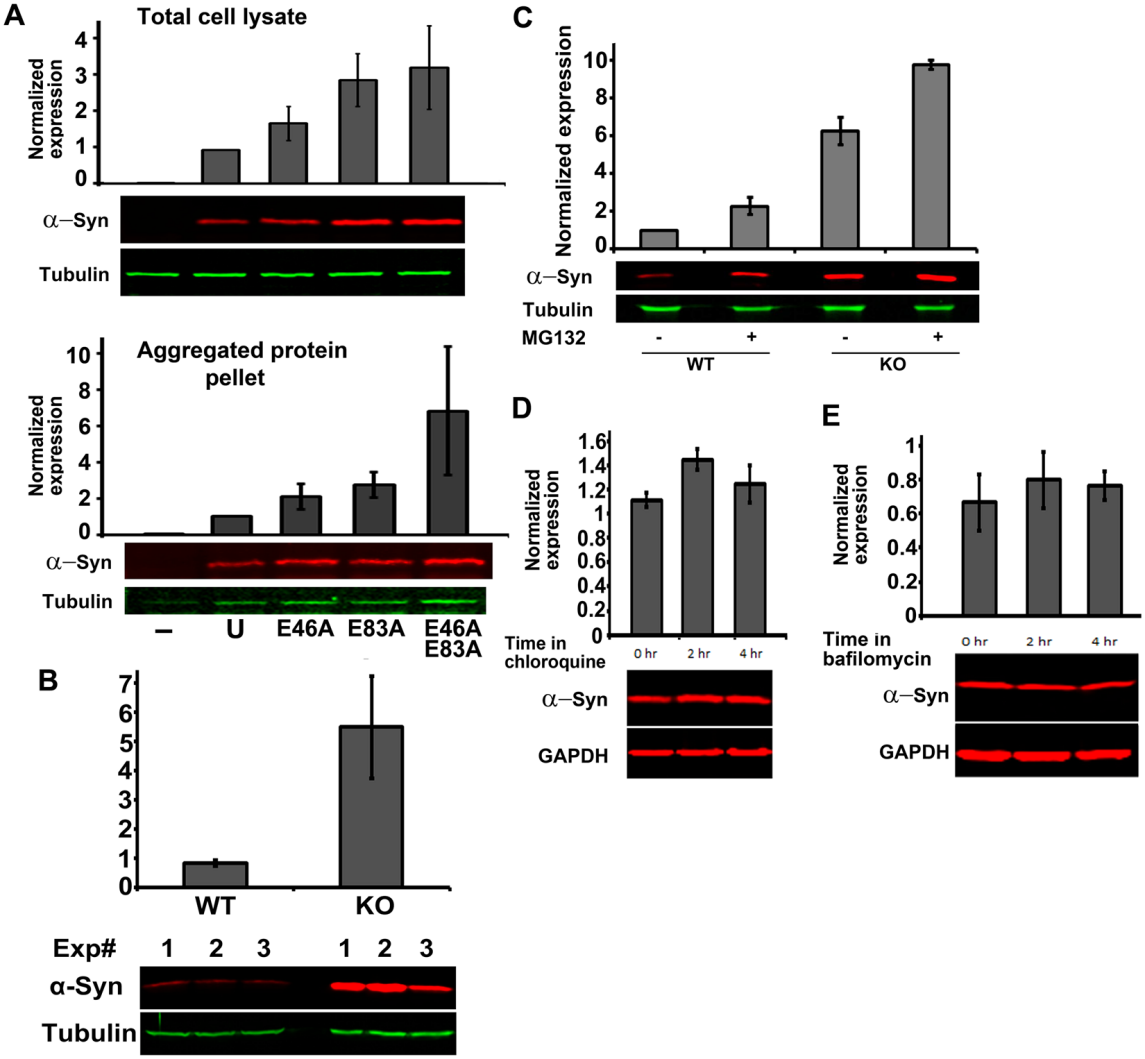
Lack of arginylation leads to α-syn accumulation in cultured cells independently of proteasome degradation and renders insensitivity to inhibitors of autophagy. (**A**) Western blot quantification of the levels of unmodified α-syn (U) and α-syn mutants E46A, E83A, and E46AE83A transfected into cultured cells and analyzed in whole cell extracts (top) and pellets (bottom). The results for quantification were normalized by qPCR and tubulin loading control. (**B**) Western blot quantification of intracellular levels of α-syn transfected into wild type and Ate1 knockout cultured mouse embryonic fibroblasts. (**C**) Western blot quantification of intracellular levels of α-syn transfected into wild type and Ate1 knockout cultured mouse embryonic fibroblasts, analyzed in the absence or presence of proteasome inhibitor MG132 (6-hour treatment). (**D**,**E**) Western blot quantification of intracellular levels of α-syn transfected into Ate1 knockout cultured mouse embryonic fibroblasts, analyzed in the absence or presence of chloroquine (**D**) or bafilomycin (**E**). Bottom rows in all panels show representative immunoblots with α-syn and the loading control. For all panels, error bars represent SEM from 2 (panel C) and 3 (panels B, D and E) independent experiments, analyzed in duplicates.

It has been previously shown that for some proteins lack of arginylation may lead to their intracellular accumulation due to impairments in their proteasomal degradation. This regulation, however, has never been shown for an intact protein that has not been proteolytically pre-processed prior to arginylation. To test whether arginylation of α-syn has any effect on its intracellular accumulation, we transfected cultured mouse embryonic fibroblasts with cDNA constructs encoding either unmodified α-syn (U), or its mutant versions with substitutions of E residues at arginylated positions to A (Ala), individually (E46A and E83A), or in combination (E46AE83A double mutant), engineered to abolish arginylation at either one or both sites (Fig. 3A). While transfected unmodified α-syn did not prominently accumulate in cells, each single mutation led to an increase in its intracellular levels. Furthermore, double mutation to produce an α-syn mutant that is unable to undergo arginylation at these sites resulted in an even bigger increase in the protein levels compared to control, indicating that these mutations likely have a cumulative effect in facilitating intracellular α-syn accumulation. This difference was even more prominent in the pellets, produced by high speed centrifugation after cell lysis to enrich for α-syn aggregates, suggesting that α-syn arginylation may induce an increase in its solubility and decrease in its aggregation. Thus, arginylation at E46 and E83 is likely responsible for maintaining the overall low intracellular levels of α-syn and lack of this arginylation leads to the increase in intracellular α-syn level and its aggregation.

To further test the relation of α-syn levels to arginylation, we transfected the unmodified α-syn construct into the matching cultures of wild type and Ate1 knockout mouse embryonic fibroblasts^10^ (Fig. 3B). Consistent with our hypothesis, Ate1 knockout cells had a much higher level of transfected α-syn compared to control, suggesting that lack of arginylation leads to α-syn accumulation and Ate1 knockout background may compound this effect.

It has been previously shown that a portion of the normal α-syn in cells is turned over by degradation via ubiquitin/proteasome^31^, a mechanism that has also been found to constitute one of the possible downstream effects of arginylation. It is also known that pathologically misfolded α-syn can be removed via lysosomal degradation and autophagy, the processes that have also been recently shown to be regulated by ATE1^32^. To test whether Ate1 knockout affects α-syn degradation, through any of these pathways, we first compared the intracellular levels of α-syn transfected into wild type and Ate1 knockout cells before and after treatment with the proteasome inhibitor MG132. This treatment led to an increase in the level of α-syn in both cell types (Fig. 3C), consistent with the fact that proteasome pathway normally participates in normal α-syn turnover. However, the extent of α-syn increase in Ate1 knockout cells was not significantly different from wild type, suggesting that α-syn accumulation in the absence of arginylation does not depend on the proteasome.

To test the effect of arginylation on α-syn clearance by lysosomal degradation and autophagy, we treated the α-syn-transfected Ate1 knockout cells with chloroquine and bafilomycin – the inhibitors of lysosomal degradation and autophagy previously shown to interfere with α-syn removal from normal cells. Remarkably, neither of these treatments in Ate1 knockout led to the expected increase in intracellular levels of α-syn (Fig. 3D), suggesting that neither lysosomal degradation nor autophagy contribute substantially to the removal of α-syn, in the absence of arginylation. To test whether both pathways are generally functional in Ate1 knockout cells, we tested the effect of these inhibitors on an endogenous target of these pathways, microtubule-associated protein light chain 3 (LC3), which normally increases in levels after both treatments. Bafilomycin appeared to be fully effective (Fig. S2, right), while chloroquine treatment did not appear to strongly affect the conversion of LC3-I to LC3-II or their levels in Ate1 knockout cells (Fig. S2, right), suggesting that chloroquine-targeted pathway may be generally impaired in Ate1 knockout cells. Control experiments confirmed that both inhibitors were working as expected in wild type cells, as evidenced by the increase in LC3-I to LC3-II conversion after both treatments (Fig. S2, left).

Thus, lack of arginylation leads to intracellular α-syn accumulation and its inability to be effectively removed by the degradation mechanisms that normally facilitate α-syn clearance from the cells.

### Arginylation reduces aggregation of pre-formed α-syn fibrils

**Figure 4:**
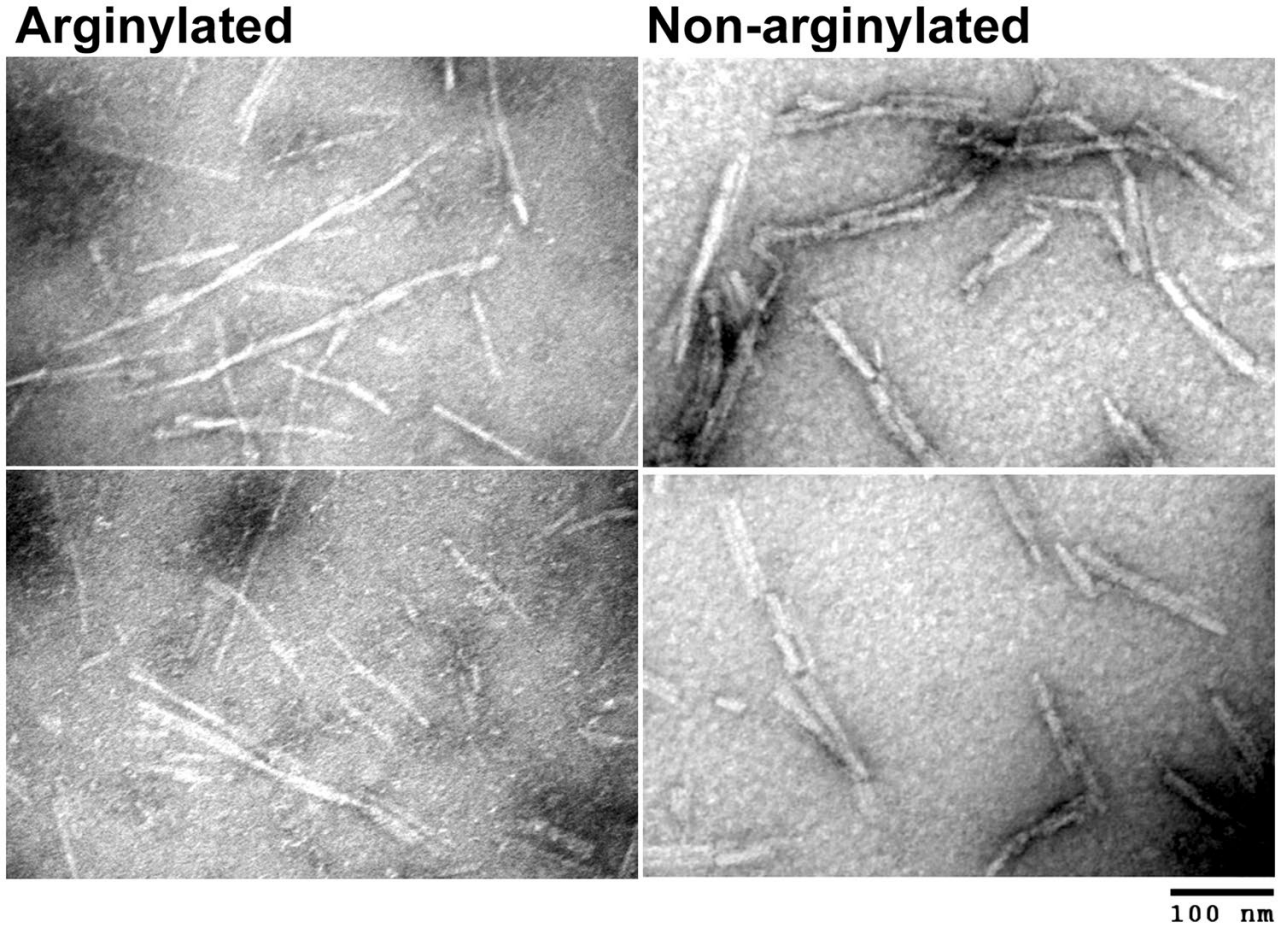
Arginylation prevents α-syn aggregation. Negative staining electron microscopy images of α-syn fibrils pre-formed from arginylated (left) and non-arginylated (right) α-syn.

To test whether α-syn arginylation affects the structural properties of α-syn fibrils, we analyzed non-arginylated and arginylated α-syn preparations, induced to form fibrils *in vitro* by prolonged shaking, using negative staining electron microscopy. Both preparations, as expected, contained large numbers of α-syn fibrils, which appeared to be individually similar to each other in width and overall length. Notably, however, the fibrils formed from non-arginylated α-syn exhibited a much higher tendency to bundle and form higher-order aggregates compared to the non-arginylated α-syn fibrils, which tended to exist more as single filaments (Fig. 4). This effect likely indicates that arginylation reduces the ability of α-syn fibrils to self-associate, a property that can potentially underlie its reduced ability to facilitate intracellular aggregation and may significantly reduce α-syn-dependent neuropathology.

### Arginylation of α-syn’s seeds reduces their ability to induce formation of intracellular aggregates in cultured neurons

To test directly whether arginylation affects the ability of α-syn fibrils to form pathological aggregates in neurons, we used a previously developed assay, in which addition of pre-formed α-syn fibrils to cultured neurons induces their uptake and leads to generation of large intracellular aggregates over the course of days^33^. We treated mouse primary hyppocampal neurons with α-syn fibrillar seeds, pre-formed *in vitro* from either non-arginylated or arginylated α-syn.

**Figure 5:**
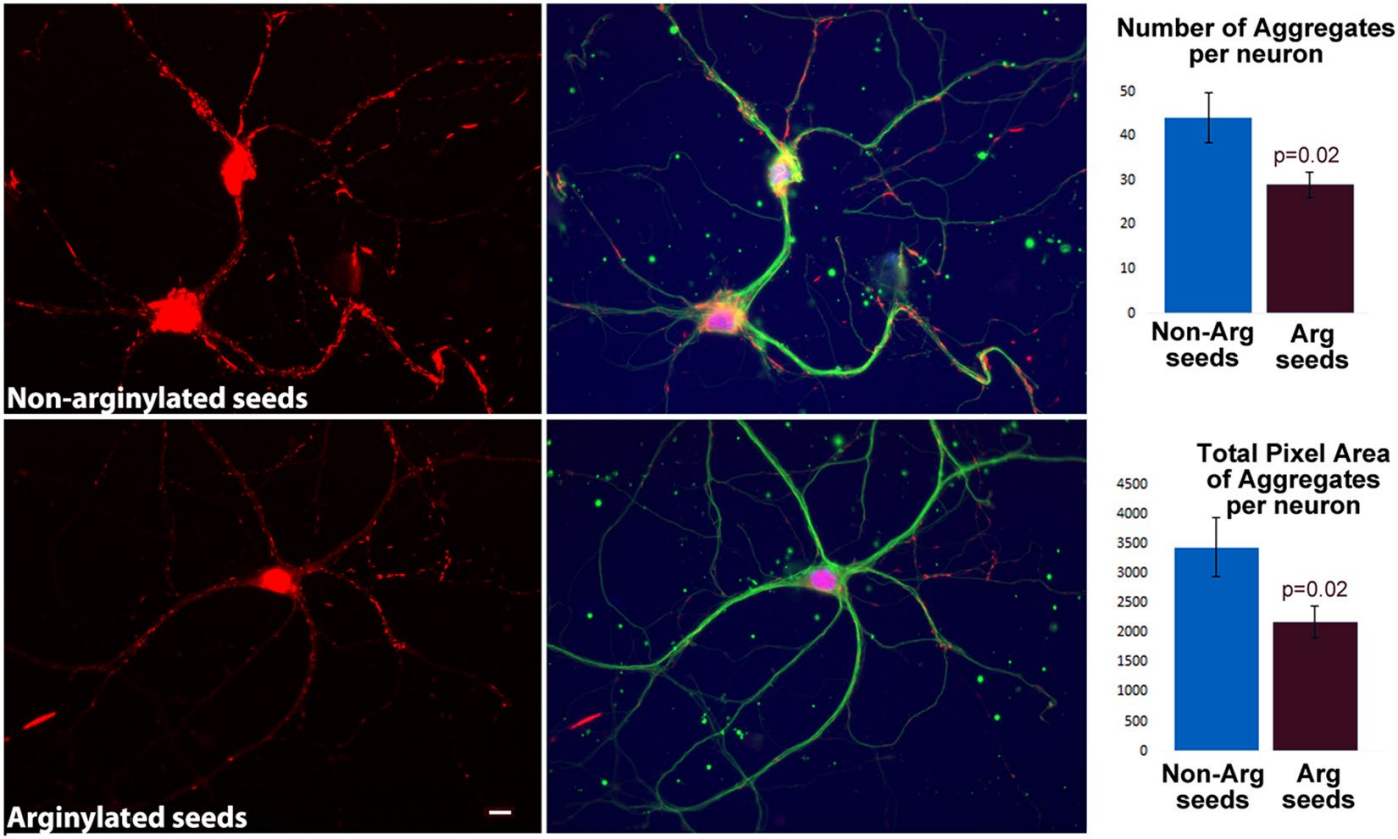
Arginylation partially prevents α-syn-induced seeding of pathological aggregates in cultured neurons. Representative images (left) and quantification (right) of α-syn aggregates formed in cultured mouse hippocampal neurons treated with α-syn seeds pre-formed from non-arginylated (top) and arginylated (bottom) α-syn. Red: α-syn pS129 staining; Green: b-III-tubulin; blue: DAPI. Non-arginylated α-syn seeds induce the formation of larger and more numerous aggregates, as seen from quantifications on the right. Error bars represent SEM of the aggregate numbers and areas quantified from 30 images obtained from 2 biological replicates and containing a total of 97 and 105 neurons treated with non-arginylated (Non-Arg) and arginylated (Arg) seeds, respectively. P values were calculated using Student’s t test. Scale bar, 10 μm.

Both types of α-syn seeds induced the formation of abundant intracellular aggregates over ~7 days, which could be prominently visualized by staining with anti- α-syn pS129 antibodies that detect the pathologic phosphorylated α-syn form (Fig. 5). To compare the effectiveness of non-arginylated and arginylated α-syn seeds in facilitating intracellular aggregate formation and growth, we quantified the total number of aggregates per neuron (the number that reflects the seeding capacity of pre-added α-syn fibrils, i.e., their ability to induce the initial formation of intracellular α-syn aggregates), as well as total aggregate area per neuron (the number that reflects the overall ability of pre-added α-syn fibrils to facilitate further aggregate growth). Remarkably, these quantifications revealed that neurons treated with arginylated α-syn fibrils contained an overall smaller number of aggregates, suggesting that arginylated α-syn seeds were less likely to serve as sites for new aggregate formation than non-arginylated ones. Furthermore, the total area of these aggregates was significantly smaller in neurons treated with arginylated α-syn seeds compared to non-arginylated ones. These data strongly suggest that arginylation can prevent the seeding and pathological propagation of α-syn aggregation in neurons.

### Generation of brain-specific Ate1 knockout mice

To test the downstream effects of arginylation on brain function and the potential implications of α-syn arginylation *in vivo*, we used our previously generated Ate1-floxed mouse, with critical region of the Ate1 gene flanked by LoxP sites^9, 34^ and crossed these mice with the commercially available mouse line expressing Cre recombinase under the Nestin promoter. In these mice (termed Nes-Ate1, or CKO for conditional knockout) Ate1 deletion occurs during embryogenesis in the entire nervous system.

Nes-Ate1 mice were born at the expected frequency, and these newborn mice exhibited no obvious defects in size or weight and no gross morphological changes compared to wild type. However, they appeared less active than their littermates and were often unsuccessful competing for nutrition, leading to progressively reduced body weights and higher incidences of mortality in the first three weeks. With extra care, including food supplementation and extended periods of time spent with the mother in the absence of wild type littermates, these mice were able to survive to adulthood and many of them had life spans comparable to the wild type. Such mice had somewhat reduced body weights, but were otherwise outwardly similar to wild type (Fig. S3). A slight body size reduction was also observed in heterozygous mice carrying the Nes-Cre transgene (marked Nes + in Fig. S3). Since we previously found in multiple Ate1 knockout mouse models that mice heterozygous for Ate1 are phenotypically normal (see, e.g., ref. 6), we assumed this effect to be due to the expression of the Cre transgene and in all the subsequent tests provided below these mice were tested along with wild type and averaged into the wild type group.

### Lack of arginylation results in accumulation of α-synuclein in the brain

**Figure 6:**
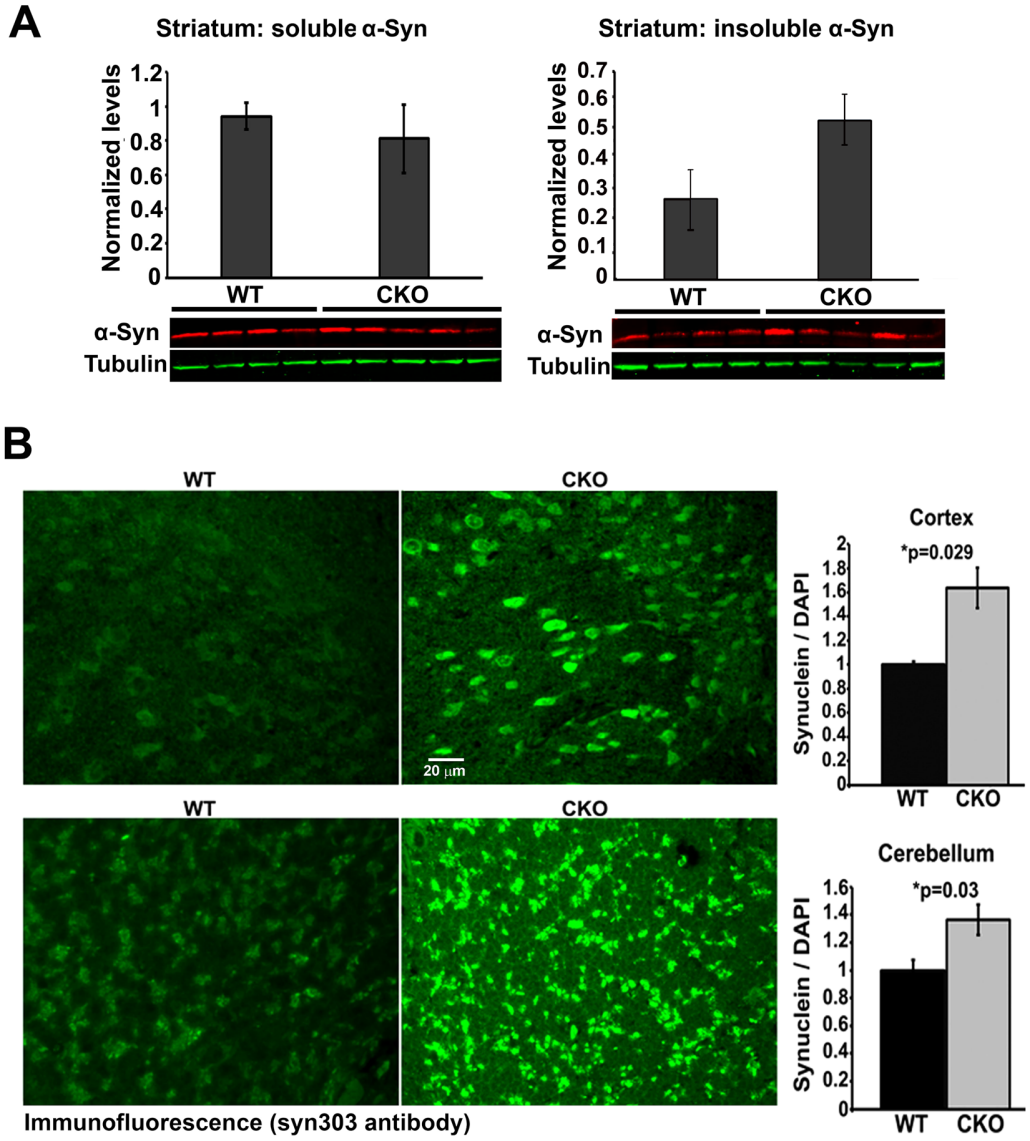
Lack of arginylation leads to α-syn accumulation in the brain. A. Western blot quantification of soluble and insoluble α-syn in the mouse striatum using α-syn 4D6 antibodies. The charts show normalized quantification of the individual repeats presented in the gels below for 8-month-old mice (4 WT and 5 CKO) adjusted by the tubulin loading control. Ratio of CKO/WT = 2.169558 is the geometric mean, p-value is one-tailed, paired Student’s T-test: P = 0.049502 (n = 3 cages). B. Representative images (left) and quantifications of the fluorescence levels (right) of the cortex and cerebellum from wild type (WT) and Nes-Ate1 (CKO) mouse brain sections immunostained with syn303 antibody.

To test whether arginylation knockout leads to increased α-syn aggregation in the brain, we performed fractionation of brain lysates (striatum) by sequential lysis that separates soluble and aggregated α-syn^35^. Quantification of the levels of α-syn in the soluble fraction revealed no significant differences between wild type and Ate1 knockout samples Fig. 6A, left). However, in the insoluble pellets, which are typically associated with disease pathology, the amount of α-syn was higher in Ate1 knockout compared to wild type (Fig. 6A, right).

We also analyzed other brain regions of these mice and found prominent accumulation of misfolded α-syn in the neuronal cell bodies in the cortex and cerebellum of Nes-Ate1 (Fig. 6B). The α-syn foci detected by this staining resembled abnormal aggregates commonly attributed to neurodegeneration in mouse models^36^. There was no increase in α-syn mRNA levels (Fig. S4). Thus, lack of arginylation resulted in intracellular accumulation and abnormal aggregation of α-syn protein in the brain.

### Ate1 knockout in the brain leads to symptoms of neurodegeneration

**Figure 7:**
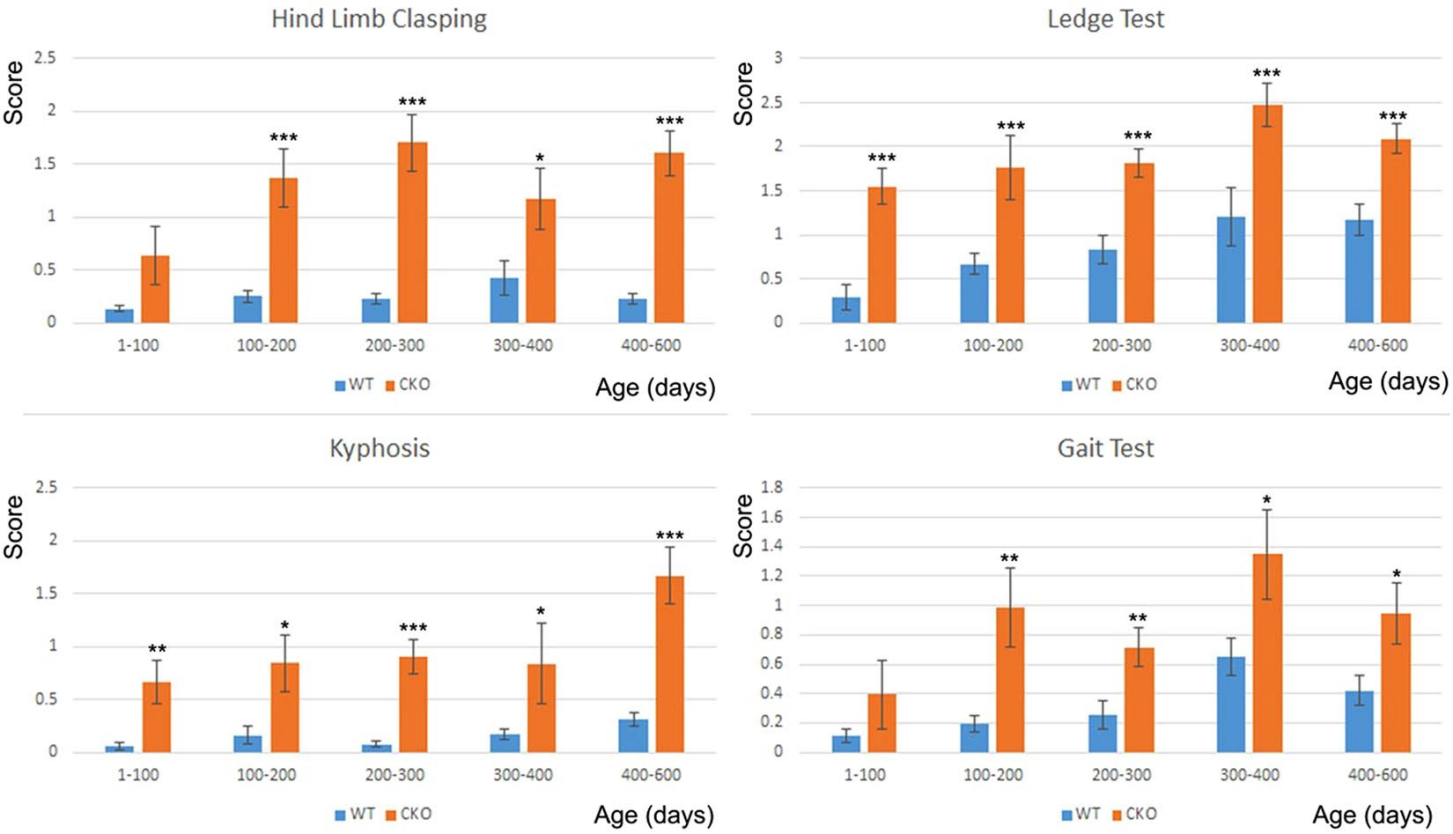
Mice with Ate1 knockout in the brain exhibit symptoms of neurodegeneration. Scores of wild type (WT) and Nes-Ate1 (CKO) mice in hind limb clasping test, ledge test, gait test, and, kyphosis test, performed as described in ref. 37 for mice of different ages. Scoring was performed blindly by seven independent investigators and averaged to obtain the final individual animal score in each test. Error bars represent SEM, n = 12 (WT, 1–100 days), 8 (CKO, 1–100 days), 21 (WT, 100–200 days), 9 (CKO, 100–200 days), 12 (WT, 200–300 days), 11 (CKO, 200–300 days), 9 (WT, 300–400 days), 6 (CKO, 300–400 days), 29 (WT, 400–600 days), and 14 (CKO, 400–600 days). P-value was determined by 1-tailed unpaired T-test. *P < 0.05; **P < 0.01; ***P < 0.001.

To test if Nes-Ate1 mice have neurological abnormalities characteristic for neurodegeneration, we performed a series of behavioral tests commonly used for evaluation of mouse models of neurodegeneration, including hind limb clasping, gait test, ledge test, and kyphosis test^37^. In these tests, mice of different ages were scored blindly by 7 independent observers that had no knowledge of the genotypes. In each test, mice received a score between 0 (normal) and 3 (severely abnormal) (see ref. 37 for the scoring criteria and Figs 7 and S5 for the data).

Nes-Ate1 mice were significantly different from control in hind limb clasping test (indicative of the neuronal impairments hallmark to a number of neurodegenerative disorders), and also differed in their performance in the ledge test (that measures motor coordination), as well as in gait and kyphosis tests (the tests that largely measure overall motor activity and brain health) (Fig. 7). These results show that arginylation knockout in the brain leads to symptoms of neurodegeneration.

## Discussion

Our study demonstrates for the first time that α-syn can be arginylated *in vivo* through a novel mid-chain mechanism that can target intact proteins *in vivo*. We find that lack of arginylation results in α-syn accumulation and aggregation and leads to the downstream effects characteristic for α-syn-driven neurodegeneration. Our results suggest that arginylation is a previously unknown mechanism that maintains normal intracellular levels of α-syn and facilitates normal brain health. This finding introduces a new level of regulation into the studies of α-syn and potentially yields a new target pathway that could lead to prevention and treatment of neurodegeneration.

Arginylation of α-syn occurs by a newly discovered mechanism that targets the acidic side chains of Asp and Glu within the intact protein chains^16^. This type of arginylation-dependent protein regulation likely carries high biological relevance, since it affects native proteins that have not been pre-processed or cleaved, however its biological relevance has not been explored until now. The current study for the first time demonstrates a direct downstream effect of side chain arginylation on a native protein target, with major implications in normal physiology and disease.

The arginylated sites on α-syn identified in our studies, E46 and E83, coincide with two previously identified sites that play important roles in α-syn function. A previous study showed that the mutation E83A facilitates α-syn intracellular aggregation and formation of amyloid-like fibers^30^. Our data suggest that this effect may be potentially related to abolishment of arginylation. Even more notably, E46 is the site of the previously identified familial α-syn mutation E46K that has been shown to underlie some of the cases of PD in human patients^38^. While it may seem that E46K mutation should at least partially mimic arginylation by introducing a positively charged residue into this normally negatively charged site, our study shows that arginylation at this site actually has the opposite effect and induces α-syn removal rather than its accumulation. Clearly, the structure of K is sufficiently different from that of R added to the side chain of E–a large, bulky, and stably positively charged entity that is expected to strongly affect the folding and molecular interactions of α-syn. We predict that at least some of the effects of the E46K mutation in PD patients are related to the fact that this mutation renders α-syn ineligible for arginylation at this site. In support, α-syn E46K mutants aggregate and accumulate in cultured cells^39^, reminiscent to the effects seen in our studies. Exploring the possible connection between E46K α-syn pathology and arginylation constitutes an exciting direction of further studies.

Our data show that arginylation prevents the pathological ability of α-syn to seed and propagate misfolded aggregates in cultured neurons. This effect is especially remarkable, given the fact that arginylated α-syn seeds added to cells differ from non-arginylated seeds by an estimated 5% of arginylated subunits, which result in over 30% downstream reduction in the aggregate numbers and area. These data strongly suggest that arginylation may affect α-syn early on, possibly right after its de novo synthesis, to give it a “good start” and ensure its correct folding and function. It is also possible that arginylation prevents α-syn propagation from cell to cell, an effect that could potentially lead to exploring α-syn﻿arginylation as a therapeutic strategy for neurodegeneration. Finally, given the fact that non-arginylated α-syn seeds are more prone to bundling, it is possible that the regularly placed Arg on the α-syn surface exert an electrostatic effect, preventing the side to side association of positively charged fibrils by a mechanism similar to that previously proposed for actin^10^. Regardless of the exact mechanism, it appears likely that arginylation constitutes a previously unknown core neuroprotective mechanism that facilitates the seeding of normal α-syn conformation in cells. This hypothesis will be elucidated in further studies.

**Figure 8:**
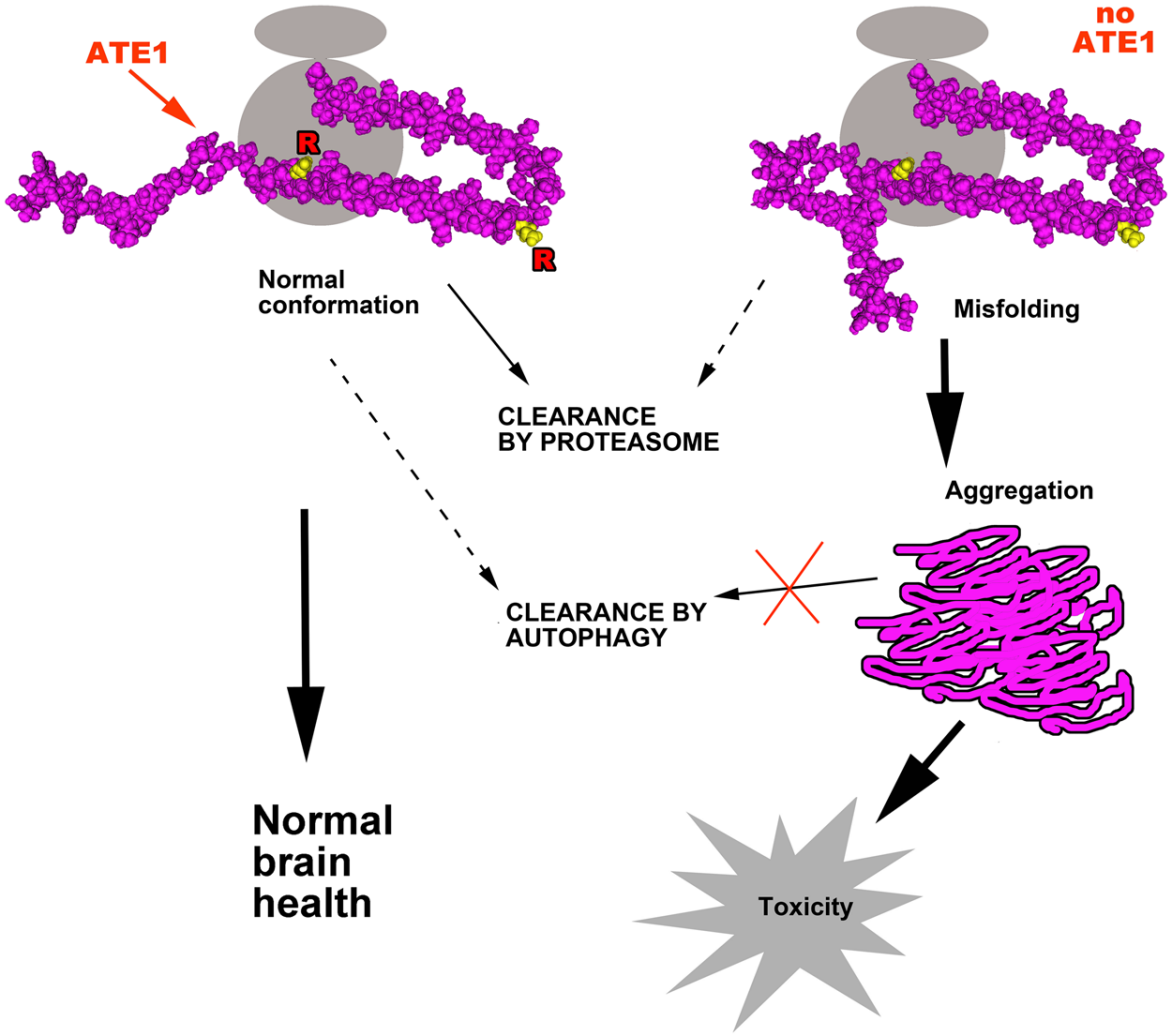
Arginylation prevents intracellular accumulation of α-syn by facilitating its normal folding and degradation. In normal cells, arginylation during, or right after translation facilitates normal α-syn folding, prevents its abnormal aggregation, and facilitates its removal. Lack of arginylation leads to early misfolding and impairment of normal degradation mechanisms, leading to propagation and accumulation of abnormally aggregated α-syn and neurodegeneration.

Previous studies showed that arginylation can target certain proteins for degradation, and that this mechanism likely acts in neurodegeneration by facilitating removal of pathologically generated protein fragments^15^. However, we find that full length α-syn arginylation does not facilitate its proteasomal degradation. Moreover, α-syn levels in Ate1 knockout cells are insensitive to the inhibitors of autophagy. We find that the two different autophagy inhibitors used in our assays, bafilomycin and chloroquine, appear to act differently in their specificity toward α-syn. Treatment of Ate1 knockout cells with bafilomycin, the classical autophagy inhibitor, affects LC3 levels as expected but does not affect the levels of α-syn, suggesting that in the Ate1 knockout background the pathway itself is still working but can no longer target α-syn. In contrast, chloroquine treatment in Ate1 knockout cells not only had no effect on α-syn, but also affected LC3 levels very little, suggesting that the pathways targeted by this inhibitor may be generally impaired in Ate1 knockout cells. This result is consistent with prior findings^32, 40^. Thus, failure of α-syn to be properly degraded in Ate1 knockout cells appears to be due to a combination of mechanisms, some of them linked to general impairments of protein degradation in these cells, others mediated directly by α-syn arginylation that, in normal cells, would prevent its misfolding and thus facilitate its normal turnover (Fig. 8).

**Figure 9:**
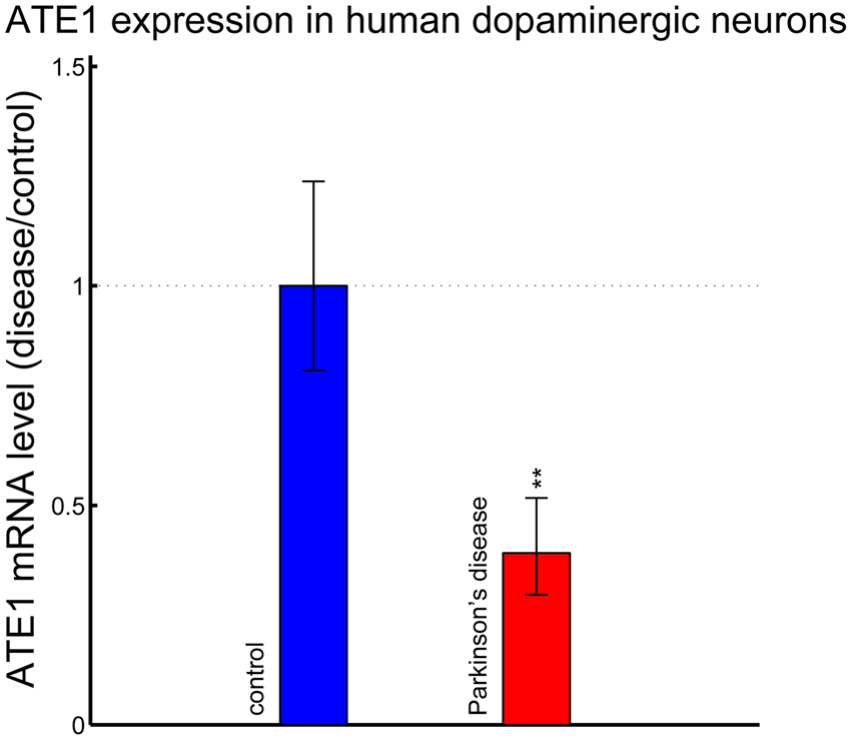
ATE1 levels are significantly lowered in dopaminergic neurons from human patients with Parkinson’s disease. Plot showing relative ATE1 expression levels in laser-captured human dopaminergic neurons in substantia nigra of normal brain and PD patients calculated from the public GSE20141 microarray data set^44^ using RMA method. Calculation was performed in log2 unit and plotted in linear fashion. p-value = 0.008.

Our previous studies suggest that arginylation can occur co-translationally via a pool of ATE1 that is associated with the ribosomes and likely targets nascent peptides during synthesis. This mechanism accounts for differential cotranslational targeting of beta and gamma actin isoforms during cell migration^41^. It is possible that co-translational arginylation of α-syn may define its structure early on, possibly as it emerges from the ribosome, thus “seeding” its normal conformation in the cell. Lack of arginylation in this case would lead to its co-translational misfolding and initiation of neurodegenerative pathology. Based on our finding that a relatively minor fraction of α-syn (~5%) is arginylated in the steady state, we hypothesize that this fraction may serve as a seed for the normal structure of emerging α-syn molecules, and that loss of this seeding mechanism leads to misfolding and impaired degradation, eventually resulting in α-syn accumulation. In support, high throughput studies from other groups show that ATE1 is significantly reduced in dopaminergic neurons of human PD patients (Fig. 9), suggesting that this reduction likely correlates with reduced α-syn arginylation and neurodegeneration. Investigating this mechanism and its relation to neurodegeneration in human patients constitutes an exciting direction of future studies.

## Experimental Procedures

### Mice

Ate1-floxed mice, previously generated in our lab^9, 34^ were crossed with commercially available mouse line expressing Cre recombinase under Nestin promoter (Jackson Laboratory strain B6.Cg-Tg(Nes-cre)1Kln/J). Mice were bred and maintained in a mixed C57Bl6/129CVJ background. All experiments were performed in accordance with relevant guidelines and regulations, according to the protocols approved by the University of Pennsylvania IACUC committee.

### Mouse behavior scoring

Evaluation of neurodegenerative symptoms in mice was performed as described in ref. 37. For hind limb clasping, each mouse was picked up by the tail and held for 3 10-sec intervals to observe hind limb position and movement. For gait and kyphosis test, each mouse was placed on a horizontal surface and allowed to move freely for ~30 sec-1 min to observe the posture and the presence and severity of a hump on the back. For ledge test, each mouse was placed on the edge of an open cage and observed as it moved forward to reach the far end. All these tests were video recorded and shown simultaneously to a group of seven investigators who recorded their scores without the knowledge of the genotypes. For each animal, scores for all seven investigators were averaged and plotted as a function of age.

### Antibodies

α-syn mouse monoclonal syn303 antibody was a generous gift from Dr. V. M. Lee (dilutions: 1:200 for immunofluorescence and 1:2000 for Western blotting). Mouse monoclonal Anti-alpha Synuclein antibody [4D6] (dilution: 1:2000 for Western blotting), rabbit monoclonal [EP1536Y] to alpha synuclein (phospho S129) (dilution: 1:250 for immunofluorescence), and mouse monoclonal antibody [6C5] to GAPDH (dilution: 1:5000 for Western blotting) were purchased from Abcam. Rabbit polyclonal Anti-p62 (SQSTM1) was purchased from MBL. Mouse monoclonal anti- β -III tubulin was from R&D Systems (MAB1195, dilutions: 1:100 for immunofluorescence), β Tubulin antibody (H-235) was from Santa Cruz Biotechnology (sc-9104, dilution 1:3000 for Western), rabbit polyclonal LC3B antibody against LC3 was purchased from Cell Signaling. As the secondary antibodies, we used IRDye® 800CW Goat anti-Rabbit IgG (H + L) (926-32211 LI-COR Biosciences), and IRDye® 680RD Goat anti-Mouse IgG (H + L),(926-68070 LI-COR Biosciences) for Western blotting (1:5000 dilution), and Alexa Fluor 488 donkey anti-rabbit IgG (A21206 Invitrogen), Alexa Fluor 488 goat anti-mouse IgG (A11001 Invitrogen), Alexa Fluor 594 donkey anti-mouse IgG (A21203, Invitrogen), Alexa Fluor 594 donkey Anti-rabbit IgG (A21207, Invitrogen) for immunofluorescence (1:1000 dilution).

### Plasmids

For cloning of mouse α-syn, mouse brain total RNA was prepared with RNeasy Mini Kit (QIAGEN Cat. 74104) and cDNA were prepared with High Capacity cDNA Reverse Transcription Kits (Applied Biosystems Cat. 4368814). Mouse α-syn ORF was amplified from mouse brain cDNA template by reverse transcription(RT)-PCR using primers mSyn F: TCTTTACATATGGATGTGTTCATGAAAGGAC and mSyn R: AATGAAGCTTTTAGGCTTCAGGCTCATAGTC. The resulting PCR product was double digested with Nde I and Hind III and subcloned into pRK172. Mouse α-syn gene was then PCR amplified from this template using primers AGGAAGCTTAGCCATGGATGTGTTCATGAAAGG (forward) and AGGATCGATTAGGCTTCAGGCTCATAGTCTTGG (reverse). The PCR product was double digested with HindIII and Cla I, and subcloned into pcDNA3.1(Life Technologies) Mouse α-syn mutations E46A, E83A, and E46A/E83A were created by the QuickChange II site-directed mutagenesis kit (Agilent Technologies, Cat. 200523) using the following primers

E46A f GTAGGTTCCAAAACTAAGGCTGGAGTGGTTCATGG

E46A r CCATGAACCACTCCAGCCTTAGTTTTGGAACCTAC

E83A f GCAGTCGCTCAGAAGACAGTGGCTGGAGCTGGG

E83A r CCCAGCTCCAGCCACTGTCTTCTGAGCGACTGC

### Protein samples

Human tau, α-syn, and TDP-43 protein samples for the initial *in vitro* arginylation experiments were a gift from Dr. V. M. Lee.

### FPLC purification of endogenous mouse α-syn

Freshly excised wild type mouse brains were flash frozen in liquid N2 and homogenized by grinding using mortar and pestle in liquid N2. The powder was thawed by addition of 1:2 w/v 20 mM Tris pH 8.0, containing protease inhibitor cocktail (P8340 Sigma-Aldrich) and 10 µM MG132, heated to 100 °C for 20 min and centrifuged at 20,000 × g for 30 min. The supernatants were dialyzed against 20 mM Tris, pH 8.0 at 4 °C overnight and fractionated using Hitrap Q column (GE Healthcare Bio-Sciences) using elution with a linear 0–0.5 m NaCl gradient. Protein concentration was determined using the Pierce™ BCA Protein Assay Kit (Pierce) with bovine serum albumin as a standard.

### Expression and purification of recombinant mouse α-syn

Escherichia coli BL21 (DE3) harboring pRK172-mSyn plasmid was cultured in LB supplemented with 100ug/ml ampicillin at 37 °C overnight. Cells were collected by centrifuging at 6,000 rpm for 30 min, then resuspended in 20 mM Tris pH 8.0 containing 1 mM PMSF. The purification was performed as described above for mouse brain preparation.

### *In vitro* arginylation of recombinant α-syn

*In vitro* arginylation assay was modified from^16, 20^. A typical reaction was performed in 100 μl volume, containing 50 mM Hepes, pH 7.5, 25 mM KCl, 15 mM MgCl2, 0.1 mM DTT, 2.5 mM ATP, 100uM L-[^14^C(U)]-Arginine (Moravek Biochemicals; 310 mCi/mmol), 50 μM tRNA-Arg from E.coli (Chemical Block), 1.35 μM RRS, 1.35 μM ATE1 and 13.5uM recombinant α-syn substrate. Reaction was mixed and incubated at 37 °C for 1 hour, followed by heating at 95 °C for 15 min, cooling down on ice for 20 min, and spinning at 13,000 rpm for 15 min in a tabletop microfuge. The supernatants were precipitated with 20% TCA followed by centrifugation at 13,000 rpm for 15 min.

*In vitro* arginylation protocol was modified to prepare arginylated α-syn for preformed fibrils added to cultured neurons. 2.5 mM L-[^13^C,^15^N]-Arginine (Pierce), 175 μM tRNA-Arg and 170uM recombinant α-syn were used in the reaction. After incubation at 37 °C for 2 hours, the reactions were heated at 95 °C for 15 min, and spin at 13,000 rpm for 15 min. The supernatants were concentrated to 5 mg/ml with Amocon® Ultra-15 centrifugal filter units (EMD Millipore). Reaction without ATE1 was used as control.

### Treatment of cultured neurons with α-syn fibrils

α-syn fibril preparation, transduction and seeding in cultured neurons was performed as described in ref. 33. For treatment with non-arginylated α-syn or enzymatically *in vitro* arginylated α-syn seeds, primary wild type mouse hippocampal neurons were obtained from the Neurons-R-Us facility at the University of Pennsylvania.

### 2D gel electrophoresis

10 µg of endogenous mouse α-syn and 8 µg of 14C- Arginine labeled recombinant α-syn were analyzed by two-dimensional electrophoresis and autoradiography, performed according to the carrier ampholine method by Kendrick Labs, Inc. (Madison, WI) as previously described^20^.

### Mass spectrometry and database searches

Sample preparation and tandem MS/MS were performed on TCA-precipitated FPLC fractions from mouse brain (shown in Fig. S1) analyzed in solution as previously described^28, 42^. Protein identification was performed with Integrated Proteomics Pipeline - IP2 (Integrated Proteomics Applications, Inc., San Diego, CA. http://www.integratedproteomics.com/) using ProLuCID and DTASelect2^28^. Spectrum raw files were extracted into ms1 and ms2 files from raw files using RawExtract 1.9.9 (http://fields.scripps.edu/downloads.php)^43^, and the tandem mass spectra were searched against NCBI RefSeq protein database (http://www.ncbi.nlm.nih.gov/refseq/, downloaded on May 17, 2010). In order to accurately estimate peptide probabilities and false discovery rates, we used a decoy database containing the reversed sequences of all the proteins appended to the target database. Tandem mass spectra were matched to sequences using the ProLuCID algorithm with 50 ppm peptide mass tolerance. ProLuCID searches were done on an Intel Xeon cluster running under the Linux operating system. The search space included all fully tryptic peptide candidates that fell within the mass tolerance window with maximum 2 miscleavages. Carbamidomethylation (+57.02146 Da) of cysteine was considered as a static modification, arginylation (+156.1011 or +166.1093) on Aspartic acid and Glutamic acid residues were considered as variable modifications.

The validity of peptide/spectrum matches (PSMs) was assessed with DTASelect2 using two SEQUEST defined parameters, the cross-correlation score (XCorr), and normalized difference in cross-correlation scores (DeltaCN). The search results were grouped by charge state (+1, +2, +3, and greater than +3) and tryptic status (fully tryptic, half-tryptic, and non-tryptic), resulting in 12 distinct sub-groups. In each one of these sub-groups, the distribution of XCorr, DeltaCN, and DeltaMass values for (a) direct and (b) decoy database PSMs was obtained, then the direct and decoy subsets were separated by discriminant analysis. Full separation of the direct and decoy PSM subsets is not generally possible; therefore, peptide match probabilities were calculated based on a nonparametric fit of the direct and decoy score distributions. A peptide confidence of 95% was set as the minimum threshold and only peptides with delta mass less than 5 ppm were accepted. The false discovery rate was calculated as the percentage of reverse decoy PSMs among all the PSMs that passed the 95% confidence threshold. After this last filtering step, we estimate that the peptide false discovery rates were below 1%. See ref. 28 for further details on manual data validation.

### Immunohistochemistry

Paraffin-embedded brain sections were deparaffinized with xylene, re-hydrated with sequential ethanol:water series (95:5, 80:20, 50:50 and 30:70), washed with water, boiled in 10 mM sodium citrate/0.05% Tween 20, pH 6.0 for 20 minutes for antigen retrieval, then blocked with 0.1 M Tris pH7.6 supplemented with 3% BSA for 1 hour at room temperature, and treated with primary antibodies diluted in 0.1 M Tris pH 7.6 supplemented with 3% BSA overnight at 4 °C. After treatment with primary antibodies, samples were washed with 0.1 M Tris, treated with fluorescent dye-conjugated secondary antibodies for 2 hours at room temperature, then stained with DAPI for 10 min. After triple washes with water, samples were embedded in Aqua Poly/Mount (Polysciences, Inc., 18606).

### Cell transfection for α-syn detection, and inhibitor treatment

Mammalian expression vector pcDNA3.1 containing α-syn and its mutants were transfected into wild type and Ate1 knockout mouse embryonic fibroblasts^10^ or 293 T cells at 70–90% confluence using Lipofectamine®2000 Transfection Reagent (Life Technologies). After 8 hrs in culture, cells were either collected directly into SDS sample buffer for Western blotting, or split into two equal dishes, grown overnight, and treated with 20 µM MG132, 100 nM of bafilomycin A1 (Sigma B1793), or 100 nM of chloroquine diphosphate (Sigma C6628), or DMSO (control).

### Western blotting

For transfected cells, after washing with PBS on ice, cells were collected into Eppendorf tubes and centrifuged at 17000 g for 5 min, then PBS was removed and the pellets were weighted and resuspended with 1:10 (w/v) of 2xSDS buffer, heated to 100 °C for 15 min, then centrifuged at 17000 g for 15 min. The supernatant was diluted with 1xSDS buffer and loaded onto 12% SDS- PAGE for Western blot. For testing for the presence of extra aggregated proteins, cells were collected as described above and resuspended in lysis buffer (50 mM Tris-HCl [pH 7.6], 150 mM NaCl, 0.2% Triton X-100, plus protease inhibitor cocktail) and kept on ice for 15 min to facilitate cell lysis. Cell lysates were then centrifuged at 50,000 g, and the pellets after this spin were dissolved in SDS sample buffer for Western blot analysis with Syn303.

Fractionation of the brain lysates into soluble and insoluble fractions by sequential Triton X-100 lysis was performed as described in ref. 35.

Protein bands were detected using the Odyssey CLx infrared scanning system and quantified with Image Studio Lite Ver3.1(Li Cor, Lincoln, Nebraska). Signal intensity was normalized by quantitative (q)PCR to evaluate the transfection efficiency and by loading controls tubulin and GAPDH for comparison of protein levels between samples.

### qPCR

Total RNA preparation from transfected cell was performed using RNeasy Mini Kit (QIAGEN, Cat# 74104). Reverse transcription was performed by using the High Capacity cDNA Reverse Transcription Kit (Applied Biosystems, AB, Cat. 4368814). Real-time PCR was performed using the SYBR-Green based detection in a Roche lightcycler 5.2 (Roche Applied ScienceQuantitative PCR cycling conditions were: initial denaturation at 95 °C for 30 sec, 60 cycles of 95 °C for 1 sec, 50 °C for 5 sec and 68 °C for 15 sec, followed by dissociation curve analysis. Each sample was run in triplicates and normalized relative to tubulin expression levels. The Comparative Ct (ΔΔCT) method was used for quantitation of relative mRNA levels.The following primers were used for qPCR

mSyn qPCR F: AGCTGGAAAGACAAAAGAGGG

hSyn qPCR F: AGCAGGAAAGACAAAAGAGGG

Syn qPCR R: CCTCCAACATTTGTCACTTGC

Tubulin F: TACCCTCGCATCCACTTCCCT

Tubulin R: CGCTTGGTCTTGATGGTGGCA.

### Image Analysis

For quantification of α-syn aggregates formed in cultured neurons shown in Fig. 5, neurons treated with arginylated and non-arginylated α-syn seeds, as well as PBS control, were photographed at 40x magnification at a uniform exposure, chosen to enable visualization of individual aggregates. Images were thresholded to the level of the brightest area of staining in PBS-treated negative control cells to eliminate most of the background due to non-aggregated protein. The area of the aggregates was measured in such thresholded images using the “integrated morphometry analysis” function of the Metamorph Imaging Software (Molecular Devices, Inc.). In each image, the total number of aggregates and the combined aggregate area (calculated as sum of all objects in the image) was and divided by the neuron number in the same image obtained by manual counting of DAPI-positive nuclei in the same field of view. Data was averaged between images as “number of aggregates per neuron” and “aggregate pixel area per neuron” to derive the columns, the error bars, and the p values shown in Fig. 3, bottom.

### Statistics

P-values were determined by one-tailed Welch’s t-test and one-tailed and two-tailed paired Student’s t-test.

## Electronic supplementary material

Supplementary Information

## References

[1] Norris EH, Giasson BI, Lee VM (2004). Alpha-synuclein: normal function and role in neurodegenerative diseases. Current topics in developmental biology.

[2] Spillantini MG (1997). Alpha-synuclein in Lewy bodies. Nature.

[3] Hurtig HI (2000). Alpha-synuclein cortical Lewy bodies correlate with dementia in Parkinson’s disease. Neurology.

[4] Balzi E, Choder M, Chen WN, Varshavsky A, Goffeau A (1990). Cloning and functional analysis of the arginyl-tRNA-protein transferase gene ATE1 of Saccharomyces cerevisiae. The Journal of biological chemistry.

[5] Rai R (2008). Arginyltransferase regulates alpha cardiac actin function, myofibril formation and contractility during heart development. Development.

[6] Kwon YT (2002). An essential role of N-terminal arginylation in cardiovascular development. Science.

[7] Kurosaka S (2012). Arginylation regulates myofibrils to maintain heart function and prevent dilated cardiomyopathy. Journal of molecular and cellular cardiology.

[8] Cornachione AS (2014). Arginylation of myosin heavy chain regulates skeletal muscle strength. Cell reports.

[9] Kurosaka S (2010). Arginylation-dependent neural crest cell migration is essential for mouse development. PLoS genetics.

[10] Karakozova M (2006). Arginylation of beta-actin regulates actin cytoskeleton and cell motility. Science.

[11] Wang YM, Ingoglia NA (1997). N-terminal arginylation of sciatic nerve and brain proteins following injury. Neurochemical research.

[12] Xu NS, Chakraborty G, Hassankhani A, Ingoglia NA (1993). N-terminal arginylation of proteins in explants of injured sciatic nerves and embryonic brains of rats. Neurochemical research.

[13] Bongiovanni G, Fissolo S, Barra HS, Hallak ME (1999). Posttranslational arginylation of soluble rat brain proteins after whole body hyperthermia. Journal of neuroscience research.

[14] Decca MB (2006). Protein arginylation in rat brain cytosol: a proteomic analysis. Neurochemical research.

[15] Brower CS, Piatkov KI, Varshavsky A (2013). Neurodegeneration-associated protein fragments as short-lived substrates of the N-end rule pathway. Molecular cell.

[16] Wang J (2014). Arginyltransferase ATE1 catalyzes midchain arginylation of proteins at side chain carboxylates *in vivo*. Chemistry & biology.

[17] Forman MS (2002). Tau and alpha-synuclein pathology in amygdala of Parkinsonism-dementia complex patients of Guam. The American journal of pathology.

[18] Galvin JE, Lee VM, Trojanowski JQ (2001). Synucleinopathies: clinical and pathological implications. Arch Neurol.

[19] Baba M (1998). Aggregation of alpha-synuclein in Lewy bodies of sporadic Parkinson’s disease and dementia with Lewy bodies. The American journal of pathology.

[20] Wang J (2011). Arginyltransferase is an ATP-independent self-regulating enzyme that forms distinct functional complexes *in vivo*. Chemistry & biology.

[21] Beharry C (2014). Tau-induced neurodegeneration: mechanisms and targets. Neuroscience bulletin.

[22] Kosik KS (1990). Tau protein and neurodegeneration. Molecular neurobiology.

[23] Lee EB, Lee VM, Trojanowski JQ (2012). Gains or losses: molecular mechanisms of TDP43-mediated neurodegeneration. Nat Rev Neurosci.

[24] Buratti E, Baralle FE (2009). The molecular links between TDP-43 dysfunction and neurodegeneration. Advances in genetics.

[25] Banks GT, Kuta A, Isaacs AM, Fisher EM (2008). TDP-43 is a culprit in human neurodegeneration, and not just an innocent bystander. Mammalian genome: official journal of the International Mammalian Genome Society.

[26] Nakashima-Yasuda H (2007). Co-morbidity of TDP-43 proteinopathy in Lewy body related diseases. Acta neuropathologica.

[27] Wong CC (2007). Global analysis of posttranslational protein arginylation. PLoS biology.

[28] Xu T, Wong CC, Kashina A, Yates JR (2009). Identification of N-terminally arginylated proteins and peptides by mass spectrometry. Nature protocols.

[29] Greenbaum EA (2005). The E46K mutation in alpha-synuclein increases amyloid fibril formation. The Journal of biological chemistry.

[30] Waxman EA, Emmer KL, Giasson BI (2010). Residue Glu83 plays a major role in negatively regulating alpha-synuclein amyloid formation. Biochemical and biophysical research communications.

[31] Webb JL, Ravikumar B, Atkins J, Skepper JN, Rubinsztein DC (2003). Alpha-Synuclein is degraded by both autophagy and the proteasome. The Journal of biological chemistry.

[32] Cha-Molstad H (2015). Amino-terminal arginylation targets endoplasmic reticulum chaperone BiP for autophagy through p62 binding. Nature cell biology.

[33] Volpicelli-Daley LA, Luk KC, Lee VM (2014). Addition of exogenous alpha-synuclein preformed fibrils to primary neuronal cultures to seed recruitment of endogenous alpha-synuclein to Lewy body and Lewy neurite-like aggregates. Nature protocols.

[34] Leu NA, Kurosaka S, Kashina A (2009). Conditional Tek promoter-driven deletion of arginyltransferase in the germ line causes defects in gametogenesis and early embryonic lethality in mice. PloS one.

[35] Mao, X. *et al*. Pathological alpha-synuclein transmission initiated by binding lymphocyte-activation gene 3. *Science* **353**, doi:10.1126/science.aah3374 (2016). 10.1126/science.aah3374 PMC5510615 27708076

[36] Giasson BI (2002). Neuronal alpha-synucleinopathy with severe movement disorder in mice expressing A53T human alpha-synuclein. Neuron.

[37] Guyenet, S. J. *et al*. A simple composite phenotype scoring system for evaluating mouse models of cerebellar ataxia. *Journal of visualized experiments: JoVE*, doi:10.3791/1787 (2010). 10.3791/1787 PMC3121238 20495529

[38] Zarranz JJ (2004). The new mutation, E46K, of alpha-synuclein causes Parkinson and Lewy body dementia. Annals of neurology.

[39] Pandey N, Schmidt RE, Galvin JE (2006). The alpha-synuclein mutation E46K promotes aggregation in cultured cells. Experimental neurology.

[40] Jiang, Y. *et al*. The arginylation branch of the N-end rule pathway positively regulates cellular autophagic flux and clearance of proteotoxic proteins. *Autophagy*, 1–16, doi:10.1080/15548627.2016.1222991 (2016). 10.1080/15548627.2016.1222991 PMC5103369 27560450

[41] Zhang F, Saha S, Shabalina SA, Kashina A (2010). Differential arginylation of actin isoforms is regulated by coding sequence-dependent degradation. Science.

[42] Wong CCL (2007). Global Analysis of Posttranslational Protein Arginylation. PLoS biology.

[43] McDonald WH (2004). MS1, MS2, and SQT-three unified, compact, and easily parsed file formats for the storage of shotgun proteomic spectra and identifications. Rapid communications in mass spectrometry: RCM.

[44] Zheng B (2010). PGC-1alpha, a potential therapeutic target for early intervention in Parkinson’s disease. Science translational medicine.

