# Supplementary material for: Protein arginylation targets alpha synuclein, facilitates normal brain health, and prevents neurodegeneration
Source: Sci Rep. 2017 Sep 12;7:11323. doi: 10.1038/s41598-017-11713-z (PMC5595787; doi:10.1038/s41598-017-11713-z)

"Protein arginylation targets alpha synuclein, facilitates normal brain health, and prevents neurodegeneration."

Supplemental Online Information.

Junling Wang[1], Xuemei Han[3], Nicolae Adrian Leu[1], Stephanie Sterling[1], Satoshi Kurosaka[1], Marie Fina[1], Virginia M. Lee[4], Dawei W. Dong[1,2], John R. Yates, III[3] and Anna Kashina[1#]

[1]University of Pennsylvania, School of Veterinary Medicine, Philadelphia, PA 19104
[2]Institute for Biomedical Informatics, Perelman School of Medicine, University of Pennsylvania
[3]The Scripps Research Institute, La Jolla, CA 92037
[4]University of Pennsylvania, Perelman School of Medicine, Philadelphia, PA 19104
[#]Corresponding author, lead contact; e-mail: akashina@upenn.edu

Fig. S1. α-syn preparation from the mouse brain. Coomassie blue staining and α-syn Western of the FPLC-purified α-syn from the mouse brain. Asterisk indicates the fraction (#5) from which the mass spectrometry data shown in this figure was obtained.

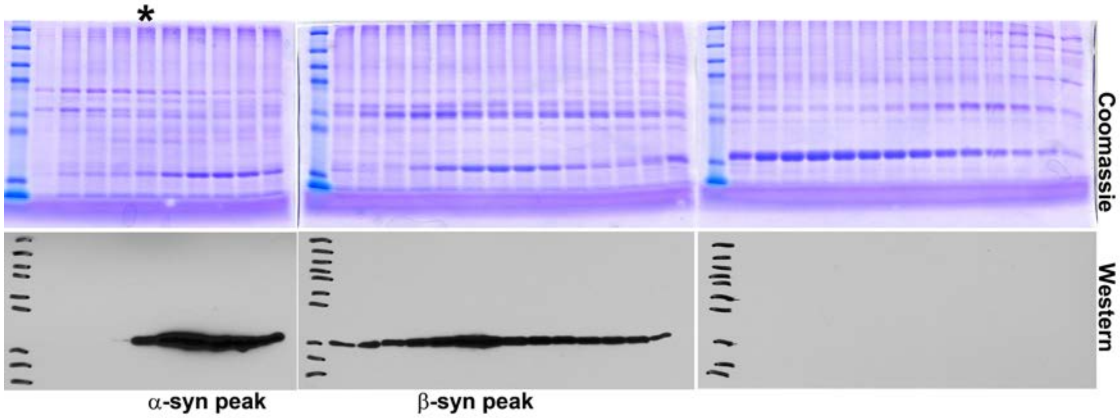

Fig. S2. Western blot quantification of the microtubule-associated protein light chain 3 (LC3) in wild type (WT) and Ate1 knockout (Ate1 KO) cultured embryonic fibroblasts treated with bafilomycin and chloroquine for different periods of time as indicated. Both inhibitors are effective in WT, but only bafilomycin has an expected effect on LC3 in Ate1 knockout. N=3.

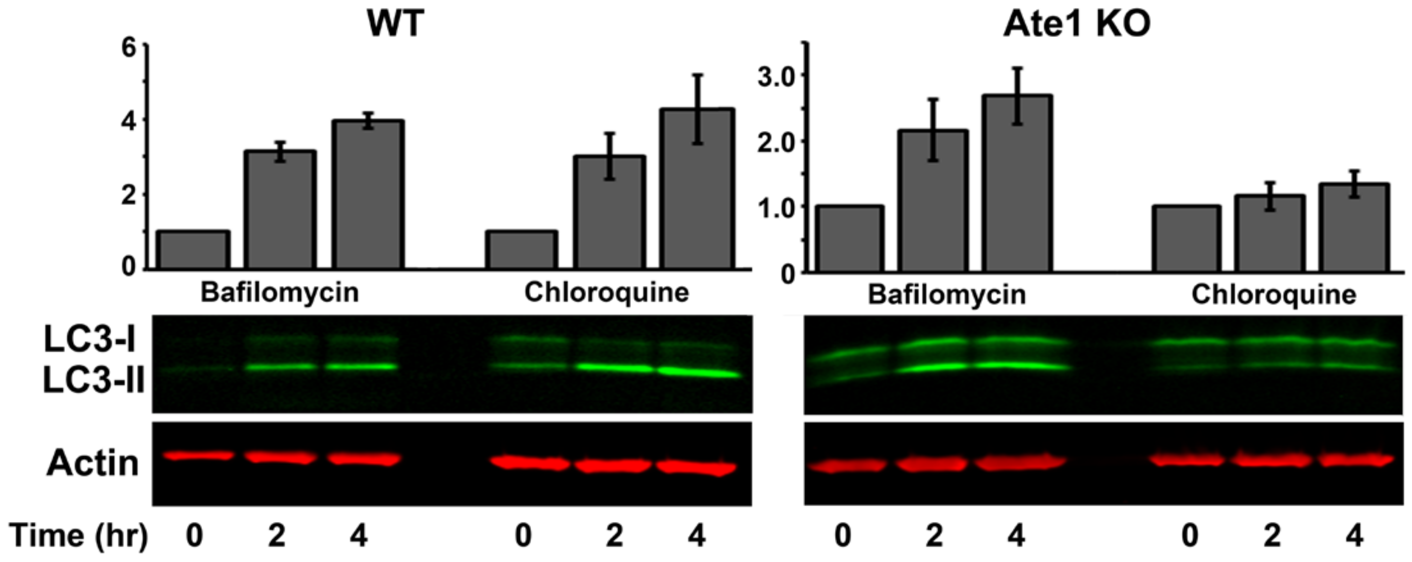

Fig. S3. Adult Nes-Ate1 mice have lower body weights, but are otherwise similar to wild type in activity and life span. Images of littermate females (top) and males (bottom). WT, wild type. CKO, Nes-Ate1. Nes+, heterozygous control carrying Nes-Cre transgene with no Ate1 deletion.

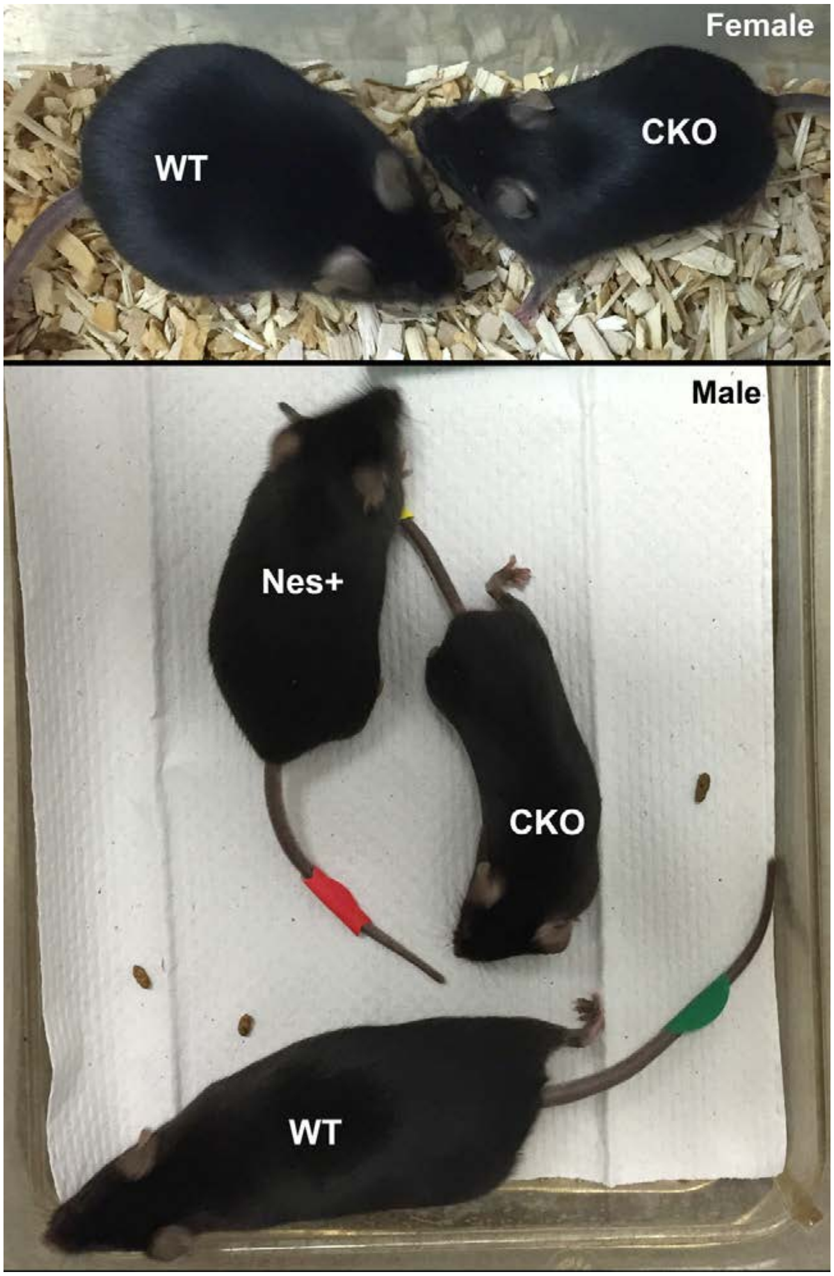

Figure S4. Expression of α, β, and γ-syn in the mouse brain does not change in Ate1 knockout. Normalized number of RNAseq 200 base paired end reads out of approximately 50M reads per sample for *Ate1*, α, β, and γ-syn (Scnca, Sncb, and Sncg, respectively), plotted as a ratio between wild type (WT) and *Ate1* knockout (CKO), averaged between 3 age-matched littermate pairs. Error bars represent SEM, n=3. P-values for each set are indicated on top, determined by the Welch's t-test.

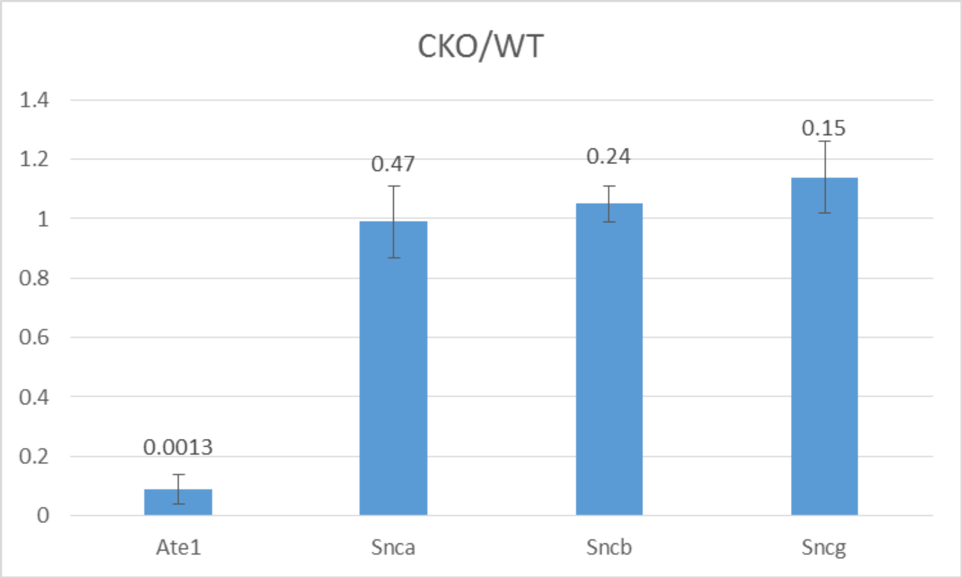

Fig. S5. Mice with Ate1 knockout in the brain exhibit symptoms of neurodegeneration. Scores of wild type and Nes-Ate1 mice in gait test, kyphosis test, ledge test, and hind leg clasping test, performed as described in (*16*) for mice of different ages and plotted against age for each animal. Scoring was performed blindly by seven independent investigators and averaged to obtain the final individual animal score in each test.

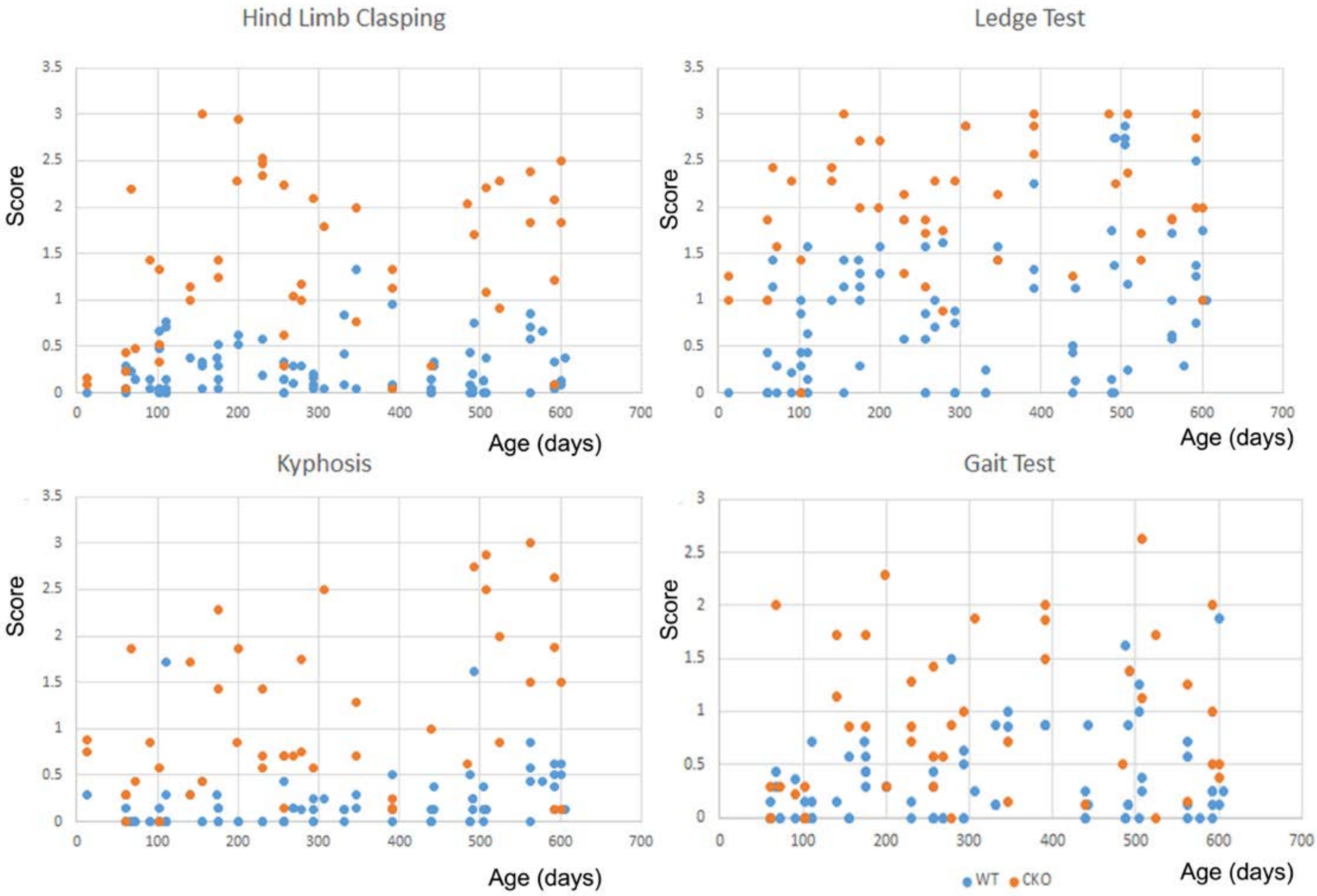

Supplemental Dataset 1: Original images used in the Western blot figures in the main text and supplement.

Fig.3A

Total cell lysate

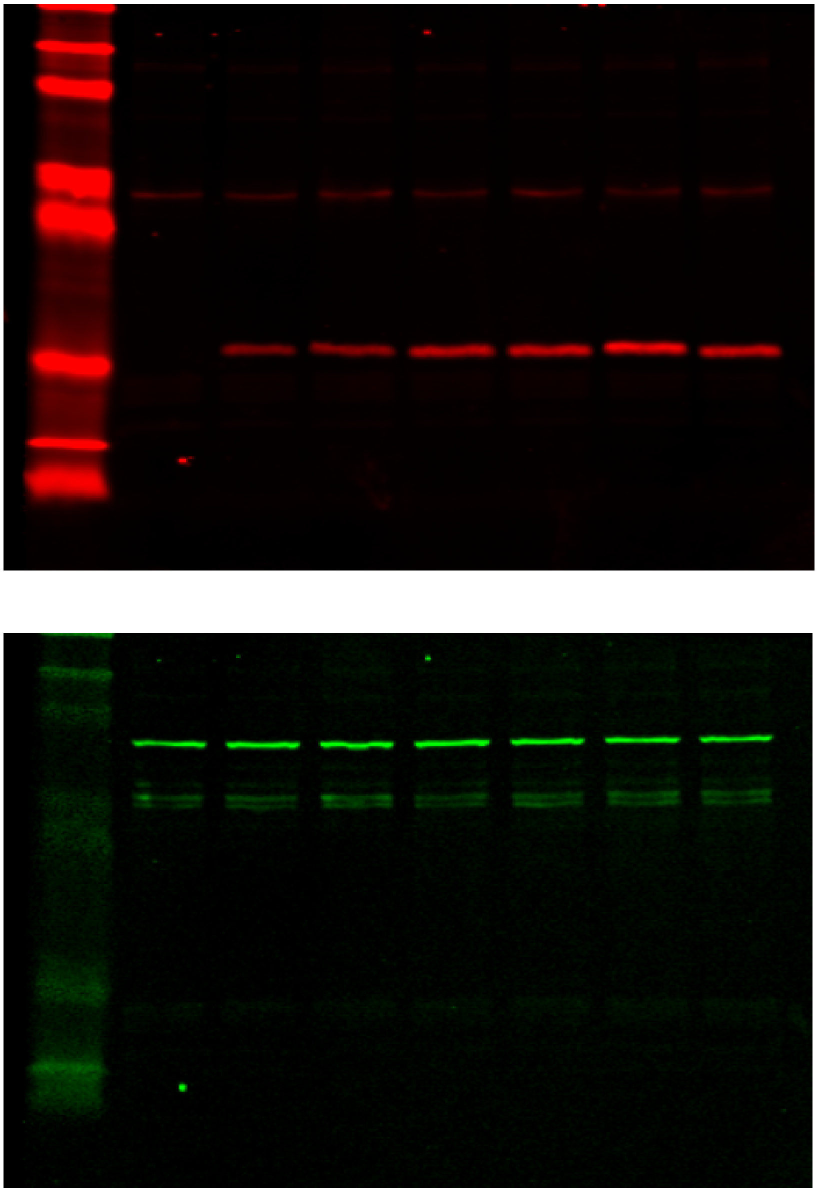

α-Synuclein

Tubulin

- U E46A E83A E46A E83A

Fig.3A

Aggregated protein pellet

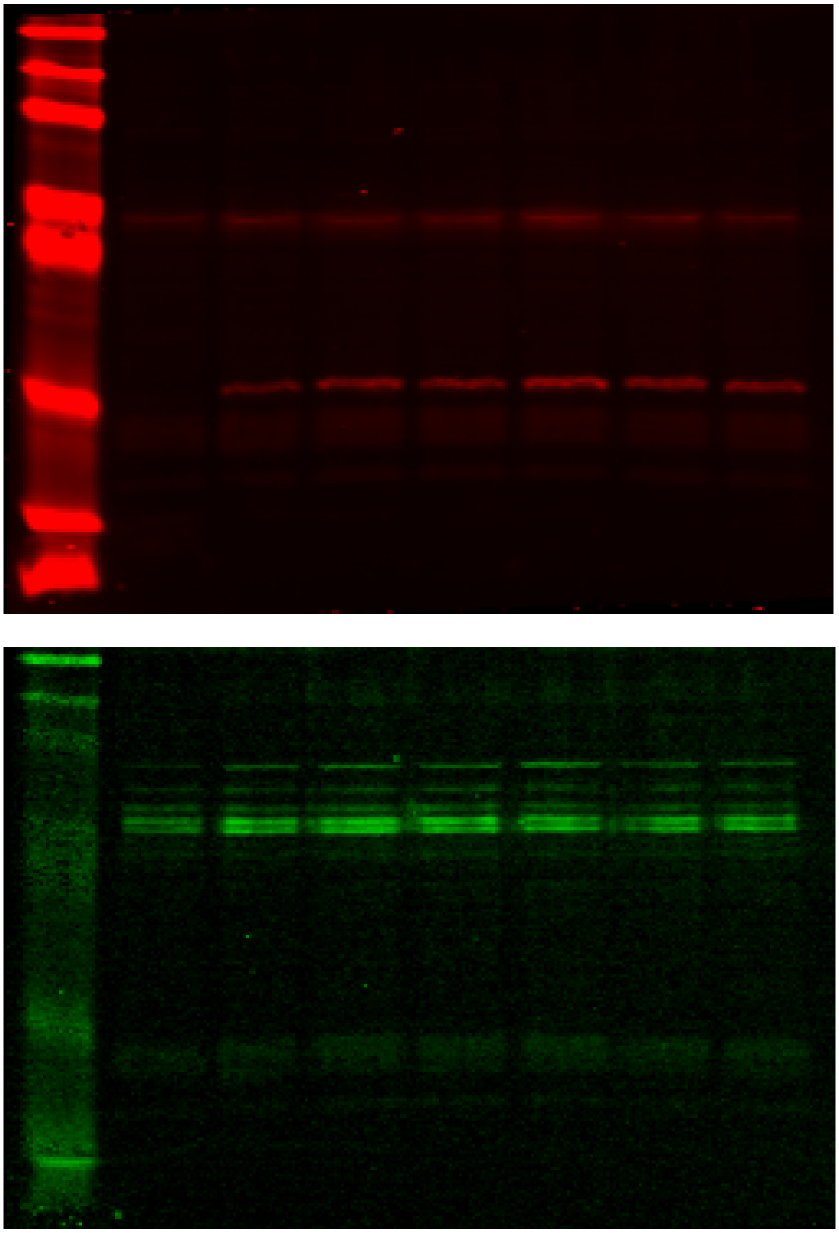

α-Synuclein

Tubulin

- U E46A E83A E46A

E83A

Fig.3B

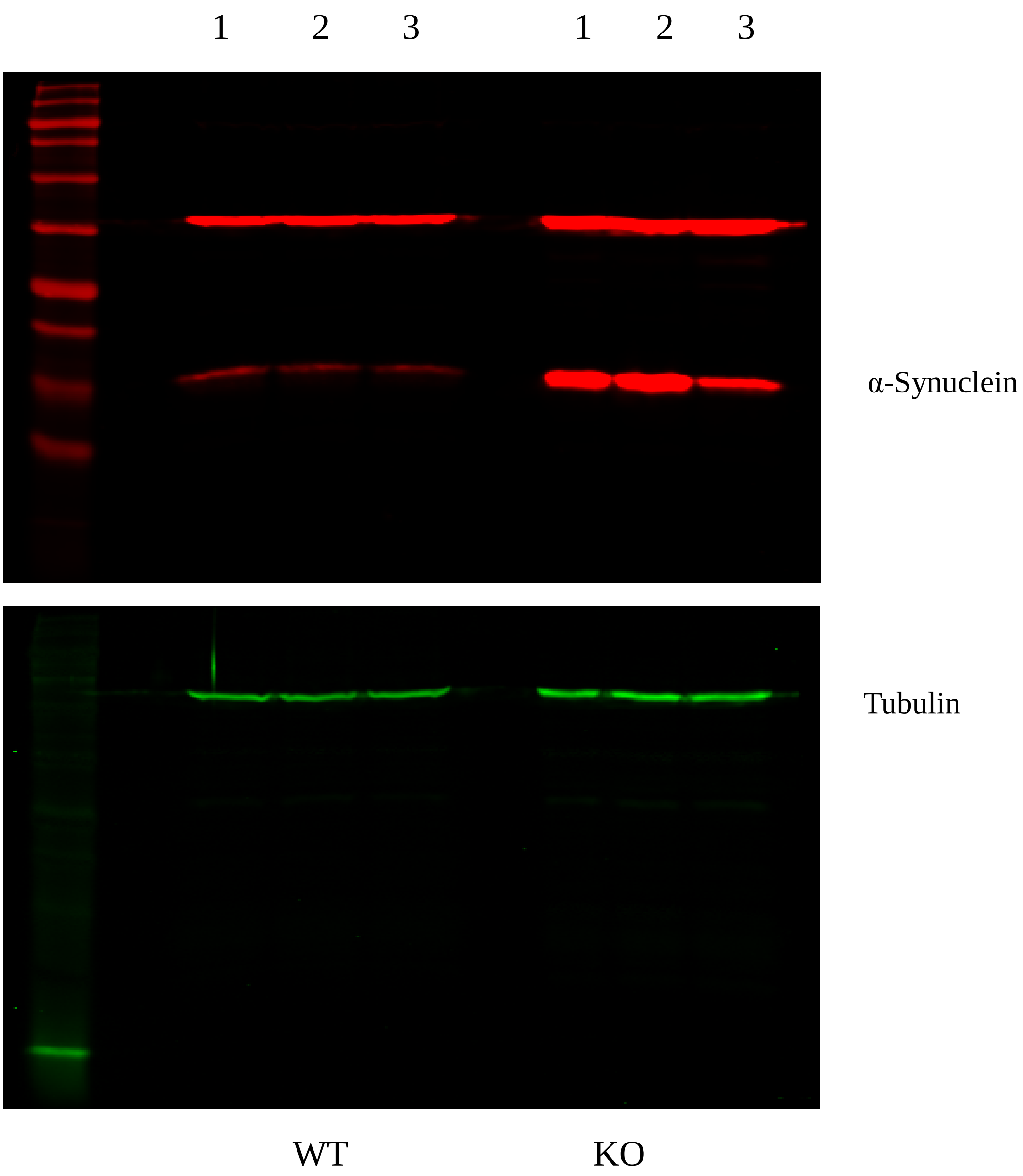

Fig.3C

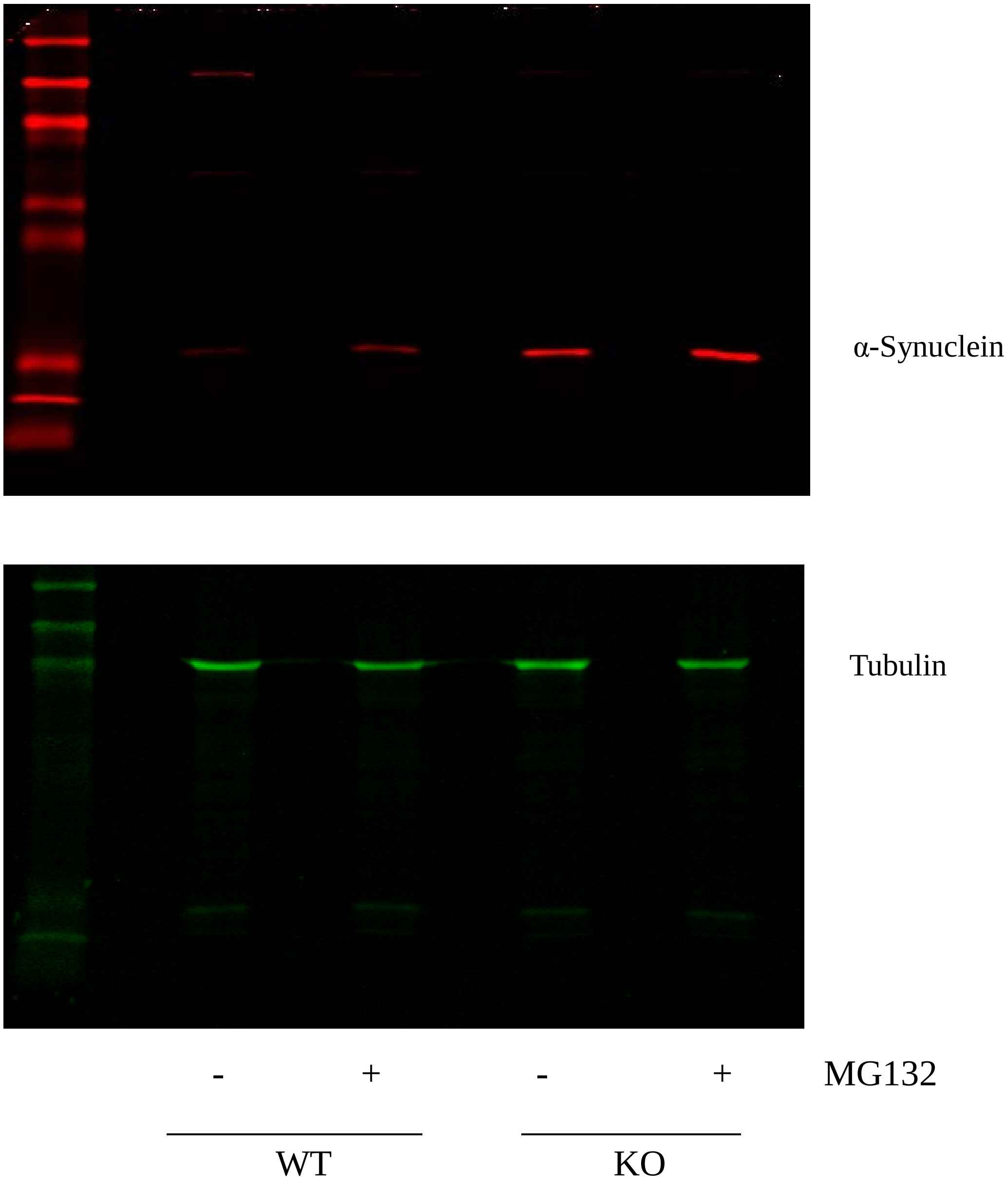

Fig.3D

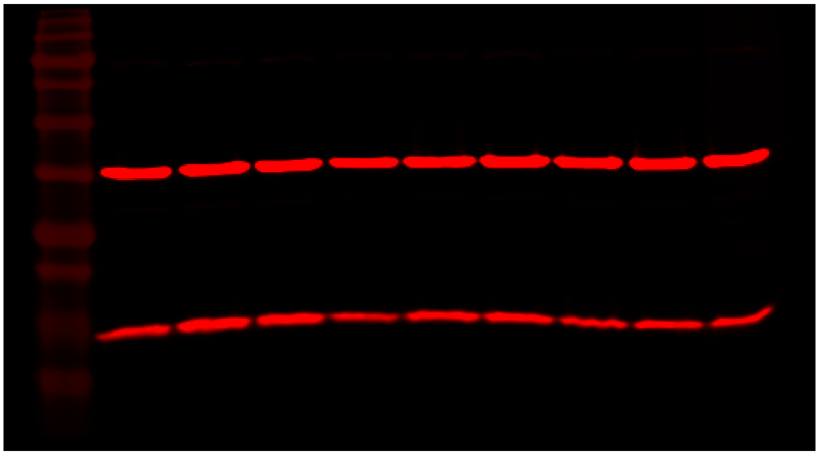
0 hr 2hr 4hr
GAPDH
α-Synuclein
Exp 1
Exp2
Exp3

Fig.3E

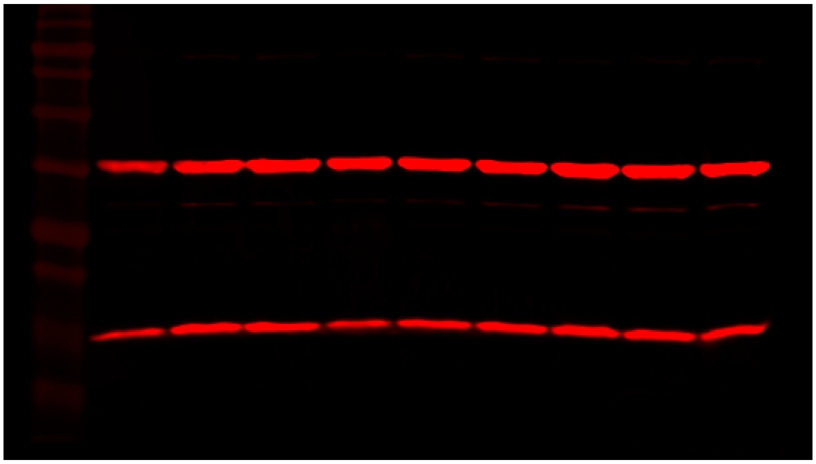
0 hr 2hr 4hr
GAPDH
α-Synuclein
Exp 1
Exp2
Exp3

Fig.6A

Striatum: soluble α-Syn

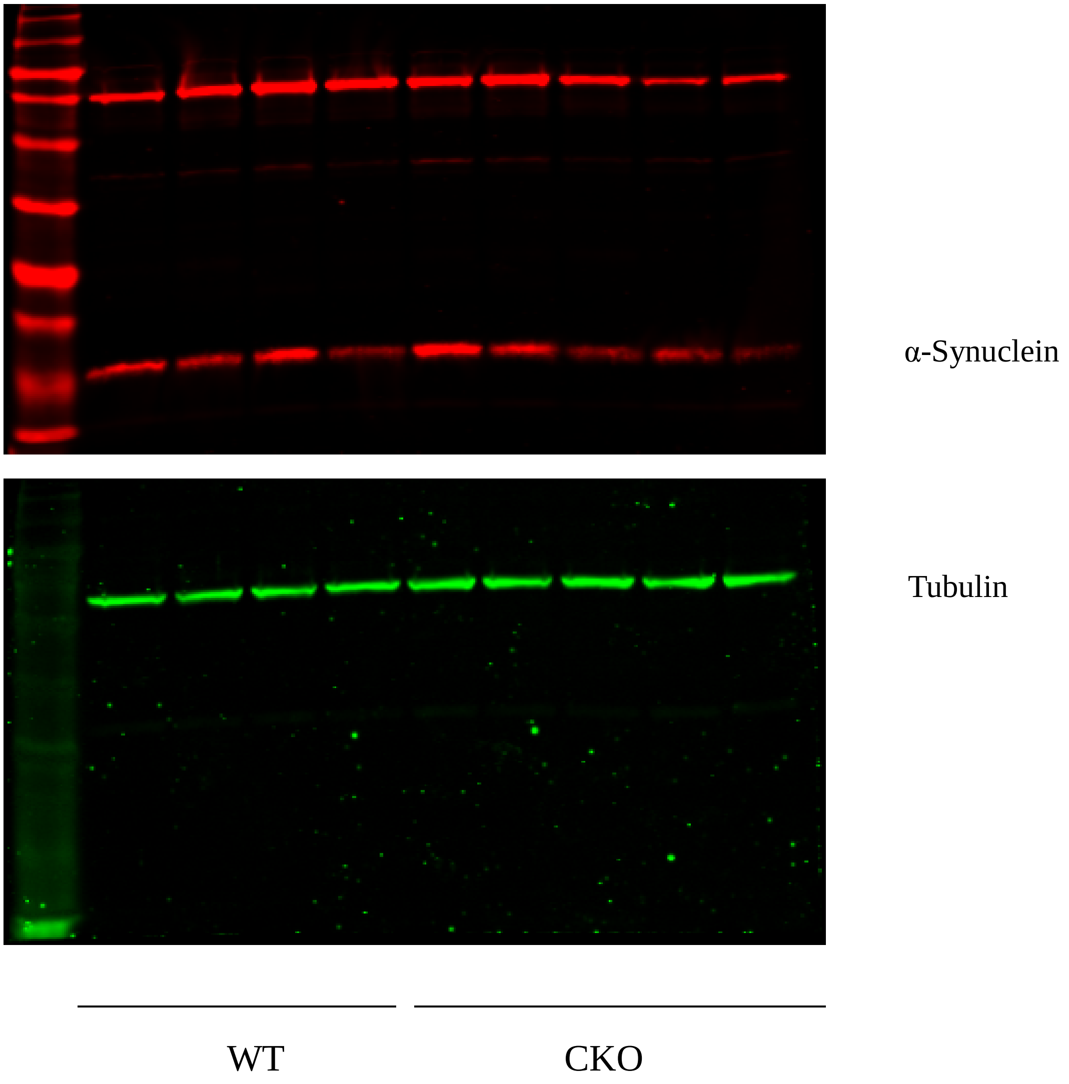

Fig.6A

Striatum: insoluble α-Syn

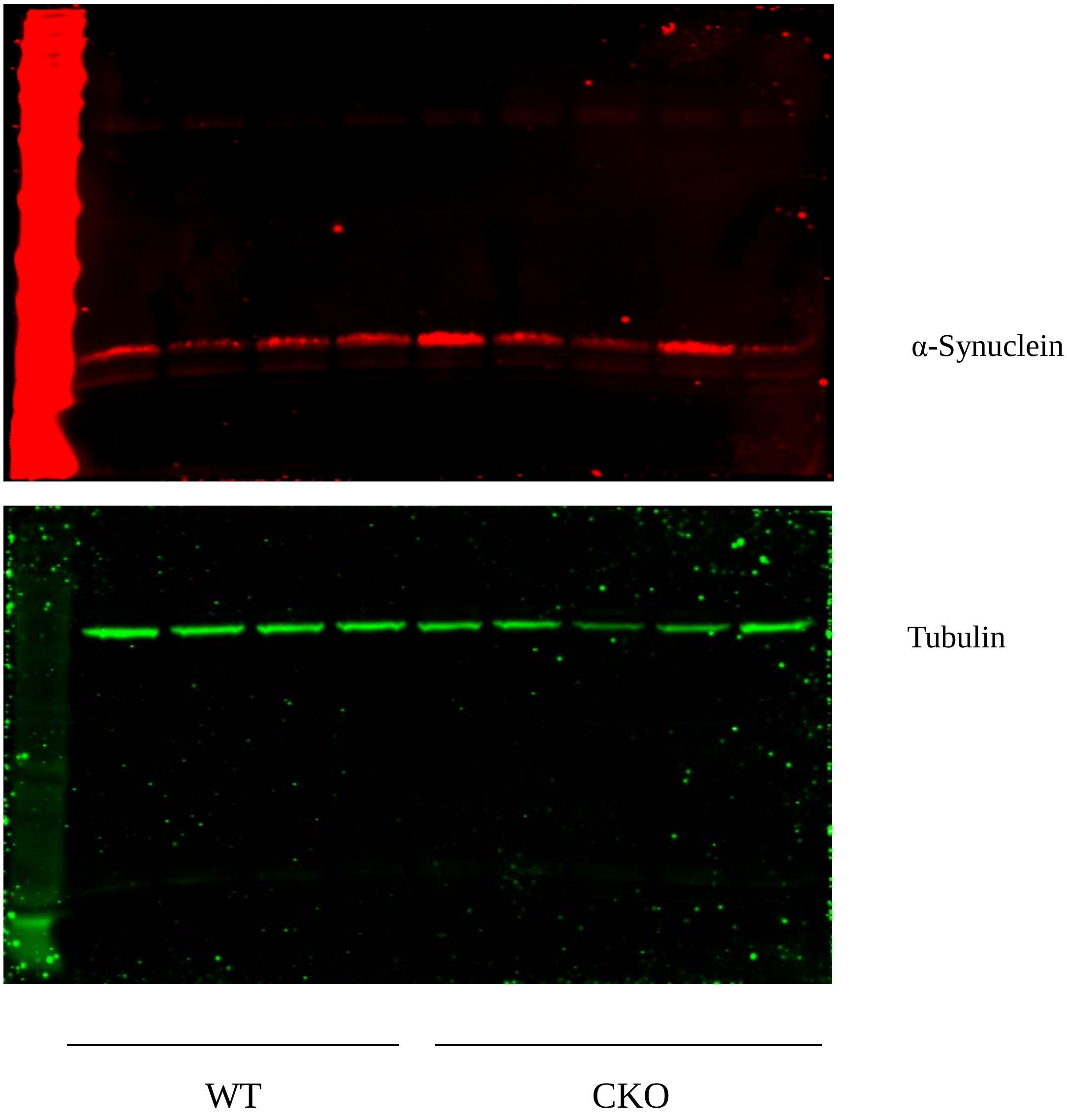

Fig.S2

WT

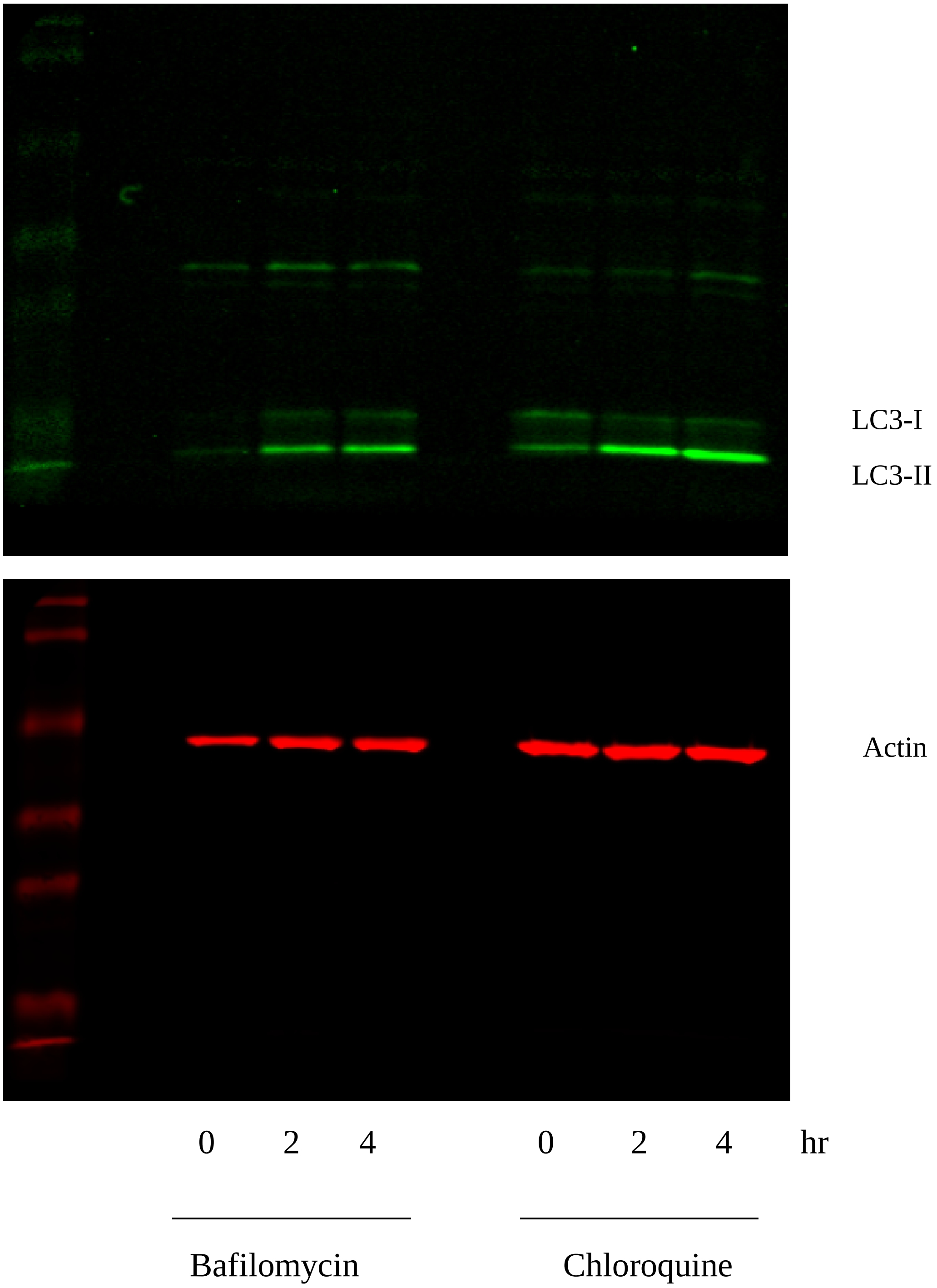

Fig. S2

Ate1 KO

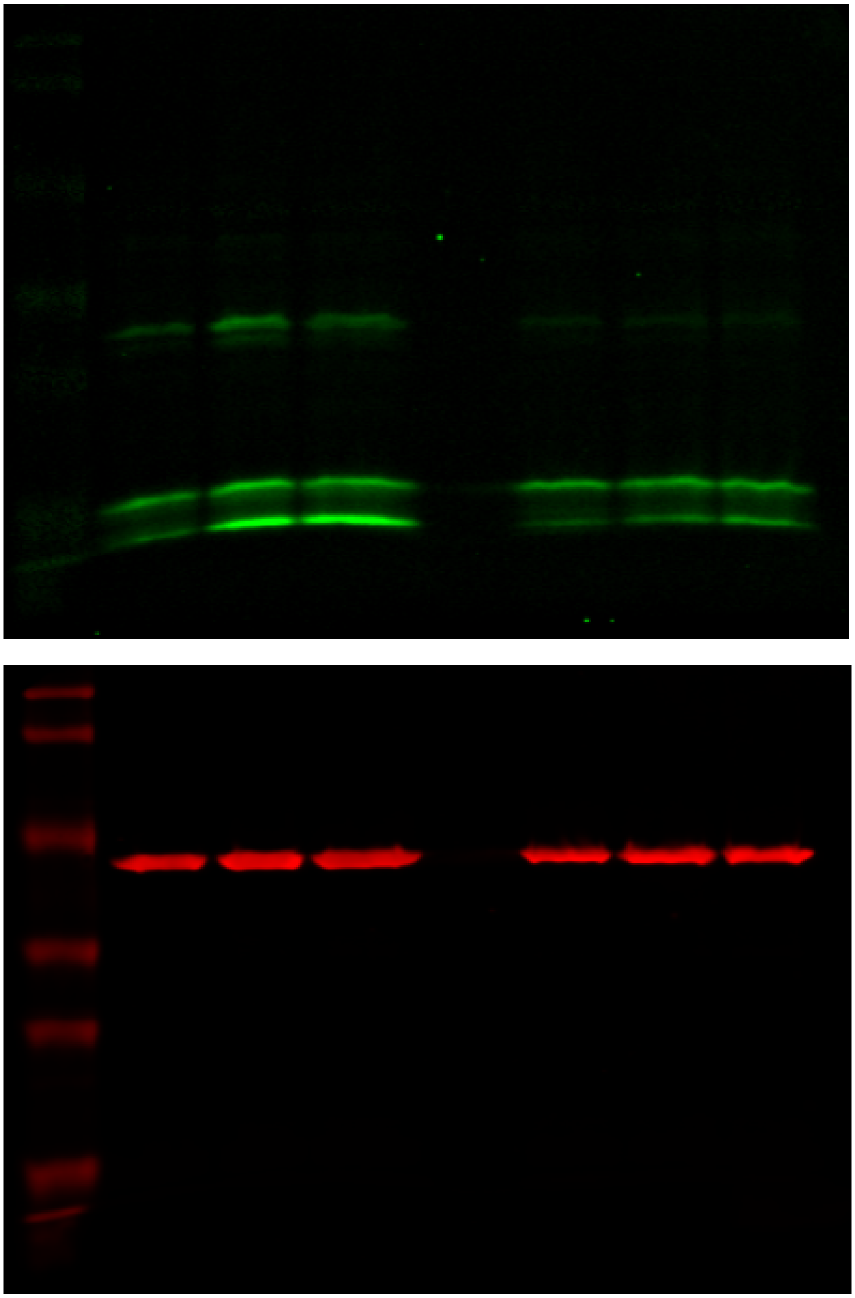

Supplement: Supplementary file 1 — Supplementary Information [file 41598_2017_11713_MOESM1_ESM.pdf]
